# Improving island occupancy predictions using ecologically informed metrics of area and isolation

**DOI:** 10.1002/ecy.70541

**Published:** 2026-10-21

**Authors:** Andrew G. Gaier, Brooks A. Kohli, Robert P. Anderson

**Affiliations:** ^1^ Department of Biology City College of New York, City University of New York New York New York USA; ^2^ Program in Biology Graduate Center, City University of New York New York New York USA; ^3^ Center for Biodiversity and Conservation American Museum of Natural History New York New York USA; ^4^ Department of Biological Sciences Ohio University Athens Ohio USA; ^5^ Division of Vertebrate Zoology (Mammalogy) American Museum of Natural History New York New York USA

**Keywords:** ecological niche model, Great Basin, insular distribution function, island biogeography, landscape connectivity, least‐cost modeling, small mammals, species distribution model

## Abstract

Understanding how species occupy islands and habitat patches remains a central question in biogeography, landscape ecology, conservation, and management, especially as natural and anthropogenic processes reconfigure habitat size and connectivity over time. The insular distribution function (IDF) provides a framework for predicting occupancy patterns for a species based on island area and isolation as proxies for extinction risk and colonization likelihood. While this model provides a conceptual bridge between island biogeography and metapopulation models, it has seen limited recent application in large part due to reliance on simplistic geometric measures of area and isolation. Here, we advance the implementation of the IDF by pulling from ecological niche modeling and landscape ecology to incorporate ecologically informed quantifications of area and isolation. We apply this framework to the montane sky‐islands of the Great Basin of the western United States, testing predictions for the Western Water Shrew (*Sorex navigator*). Compared with those based on traditional geometric metrics, the ecologically informed IDFs produced a discriminant function with lower classification error, stronger class separation, and different biological conclusions regarding the species' inferred response to isolation and area. The results from the ecologically informed IDF provide a contrast to IDF based on geographic axes and the classical interpretations for the system. Specifically, the steeper slope and lower intercept of the ecologically informed IDF point toward a system structured not so strongly by extinction‐driven relaxation, but by a more compensatory trade‐off in which both colonization and extinction have shaped occupancy patterns. Simulations of false‐absence rates (which affect datasets in lesser‐sampled systems) showed that the same inferences persisted in the presence of incomplete sampling. These results highlight the potential of integrating contemporary approaches with classic biogeographic models, as well as strengthening and clarifying links between island biogeography and landscape ecology. This updated analytical framework offers new opportunities for assessing subpopulation vulnerability, incorporating ecologically informed measures into occupancy‐based statistical frameworks, forecasting patchy species distributions across a full community, and studying how past and future environmental changes shape biodiversity patterns.

## INTRODUCTION

Natural and anthropogenic processes such as climate change and habitat degradation have had significant impacts on Earth's ecosystems (Walther et al., 2002), resulting in many species now occupying patches of island‐like habitat that vary in size and connectivity over time (Burger et al., 2019; Hodgson et al., 2012). These changes can influence the risk of local extinctions, as well as alter movement through the surrounding matrix, making it crucial to quantify and understand how species and communities respond to such alterations (Hampe & Petit, 2005). The effects of insularity on species richness were highlighted in MacArthur and Wilson's seminal equilibrium theory of island biogeography (MacArthur & Wilson, 1963), and tenets of this theory and its extensions have become cornerstones of both basic science and applications to conservation biology and natural resource management (Whittaker et al., 2023).

While the equilibrium model provides a foundation for understanding species richness in relation to island area and isolation, it does not explicitly address which particular species colonize and persist on specific islands. To extend the theory, biogeographers have explored how differential colonization and extinction among species have influenced both community‐level patterns such as nestedness (Patterson, 1987) and the distributional patterns of specific species. Notably, Lomolino (1986) developed a model for individual species that describes how area and isolation have shaped the distribution of a focal species across insular landscapes over the recent or distant past (Lomolino, 1986). The key component of this single‐species model is the insular distribution function (IDF; Figure 1), which models the expected distribution of a species on islands across a space defined by the biogeographic axes of area and isolation (Lomolino, 1999). This function is represented by a line or curve discriminating between islands where the species is present and those where it is absent. IDFs can be used to test predictions regarding the drivers of island occupancy and yield inferences regarding how a species' dispersal and persistence abilities at the population level have varied with island area, isolation, and their interaction.

**FIGURE 1:**
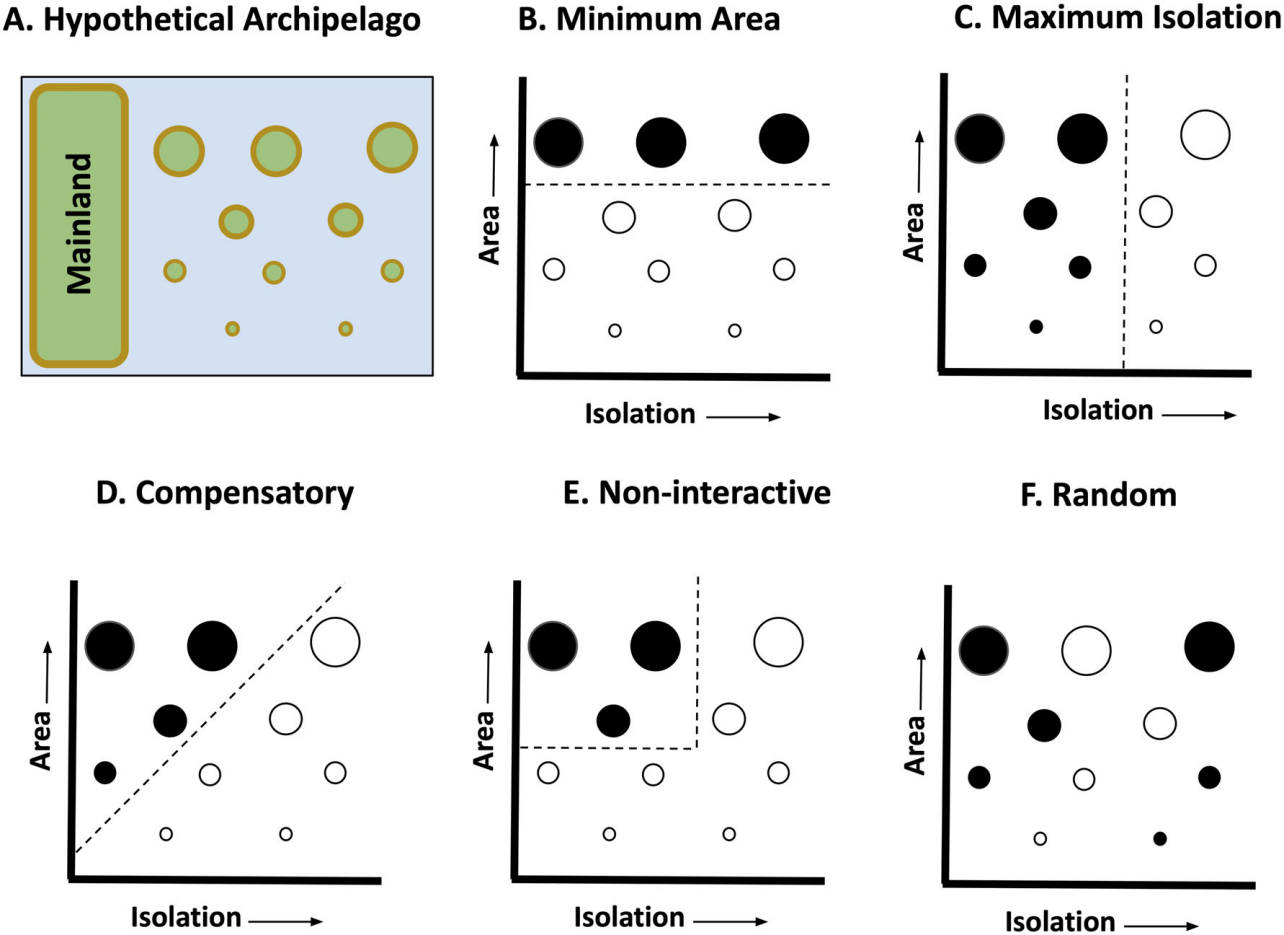
Hypothetical archipelago with islands varying in area and isolation (A), and the potential patterns of insular species distribution proposed by Lomolino (1986; B–F). By plotting island area against isolation, an insular distribution function (dashed line) is fitted to discriminate between islands where the species is present (closed circles) versus absent (open circles). (B) Minimum‐area pattern, where occupancy depends on a minimum‐area threshold. (C) Maximum isolation pattern, where occupancy depends on a maximum isolation threshold. (D) Compensatory pattern, where occupancy depends on the interaction between area and isolation. (E) Non‐interactive pattern, where occupancy depends on the additive effects of area and isolation. (F) Random pattern, where occupancy is not affected by area or isolation.

In parallel with these developments within island biogeography concerning species‐specific occupancy patterns, ecologists began developing metapopulation models that explore how similar processes influence species distributions across patchy landscapes over shorter time frames. Unlike IDFs, which describe occupancy patterns through a presumed deterministic relationship with area and isolation, metapopulation models are stochastic; they explicitly model probabilities of ongoing local immigration and extinction processes, typically using a combination of demographic and environmental factors including patch area and isolation (Hanski, 1994; Levins, 1969). Whereas metapopulation models examine occupancy dynamics over ecological time based on ongoing immigration and extinction, IDFs use present‐day occupancy patterns to infer the processes that have driven distributions over longer timescales (Gyllenberg & Hanski, 1997; Lomolino, 1999). Despite the conceptual and practical value of IDFs, their use and development have largely stalled in recent decades, in contrast to the active advancements in metapopulation ecology and its multispecies metacommunity extensions (Whittaker et al., 2023).

Concomitant with development of metapopulation and metacommunity models, landscape ecology emerged as a major area focused on understanding how environmental heterogeneity influences ecological processes across space (Wiens et al., 1993). Advances in spatio‐environmental data availability, remote sensing, GIS, and ecological niche modeling made it increasingly possible to characterize landscapes quantitatively and at biologically meaningful resolutions (Fletcher & Fortin, 2018). Rather than binary systems composed of patch and matrix, landscape ecology offers methods to provide and analyze continuous characterizations of environmental heterogeneity (Wagner & Fortin, 2005). Many advances from landscape ecology have subsequently influenced related fields such as metapopulation ecology (Howell et al., 2018) and landscape genetics (DiLeo & Wagner, 2016). However, they have rarely been incorporated into classical island biogeographic models or to IDFs at all (Whittaker et al., 2023).

These advancements highlight a longstanding but now surmountable limitation throughout much of island biogeography: acquiring ecologically realistic measurements of area and isolation for insular populations (Lawlor, 1998; Lomolino et al., 2017). Insular systems have traditionally been treated as binary landscapes, with homogenous matrix conditions and all islands equally suitable per unit area (Murphy & Lovett‐Doust, 2004). Isolation, which classically has been measured as the Euclidean distance between an island and its source, can instead be thought of as the degree of environmental resistance encountered by individuals or populations of a species that encounter or cross the intervening matrix (Leibold et al., 2004). This is especially pertinent for habitat islands where the heterogeneity of the matrix is far more pronounced than those surrounded by water. Similarly, a species' niche may limit the amount of area actually available to it within a given island (Brown, 1971), with different suitable portions likely varying in quality (Soberón & Osorio‐Olvera, 2023). Indeed, in metapopulation ecology estimates of patch quality and area have been linked with demographic data in what is often termed “patch capacity” (Cadet et al., 2003; Foltête & Vuidel, 2017). Addressing the inadequacies of binary characterizations by incorporating ecologically informed continuous metrics into IDFs represents a crucial next step in improving the predictive power of these and other biogeographic models.

Fortunately, recent advances in ecological niche models (ENMs) and connectivity modeling allow production of ecologically informed metrics of area and isolation. By estimating and mapping environmental suitability for a species across a study region, ENMs can yield informed delimitations of areas holding suitable environmental conditions—as well as their quality. Additionally, metrics for quantifying landscape connectivity can provide analogs to isolation by Euclidean distance, ripe for use with continuous maps of suitability generated by ENMs. In particular, least‐cost path (LCP) analysis (Adriaensen et al., 2003) has become a common approach for finding the path between points with the least accumulated environmental resistance. Hence, continuous maps of suitability can lead to measurements of the quality of environmental conditions on an island for a species and the permeability of the surrounding matrix with respect to that species' niche (Keeley et al., 2021).

Therefore, we aim to enhance the axes of the IDF by providing ecologically informed quantifications of island area and isolation. As a demonstration, we revisit a classic system of habitat islands: montane sky‐islands of the Great Basin in the western United States, testing this framework on a species of montane shrew. Here, we focus on a single species as a proof of concept and to establish a foundation for future extensions. Specifically, we ask: (1) Do ecologically informed quantifications of area and isolation yield an IDF with higher predictive accuracy and stronger class separation than simple geometric quantifications, and (2) Do biological conclusions differ between IDFs when using these quantifications?

## METHODS

### Study system

#### Great Basin sky‐islands

The Great Basin is a lowland drainage basin in the western United States situated between the Sierra Nevadas to the west and the Utah Rocky Mountains to the east (Figure 2). This dry, low‐elevation landscape is irregularly interrupted by isolated mountain peaks, or sky‐islands, distributed throughout the region. These sky‐islands host climates and vegetation similar to the larger adjacent ranges (Sierra Nevadas and Utah Rockies; the “mainlands”). The mammalian faunas of these islands are a subset of the larger regional pool of montane species found on the two mainlands, with particular species showing intriguing patterns of occupancy across some but not all islands (Brown, 1971; Grayson & Livingston, 1993; Lawlor, 1998). The Great Basin sky‐islands have been central to the development of insular biogeography, contributing to key concepts such as faunal relaxation (Brown, 1971), nested subsets (Patterson & Brown, 1991), and the IDF (Lomolino, 1986).

**FIGURE 2:**
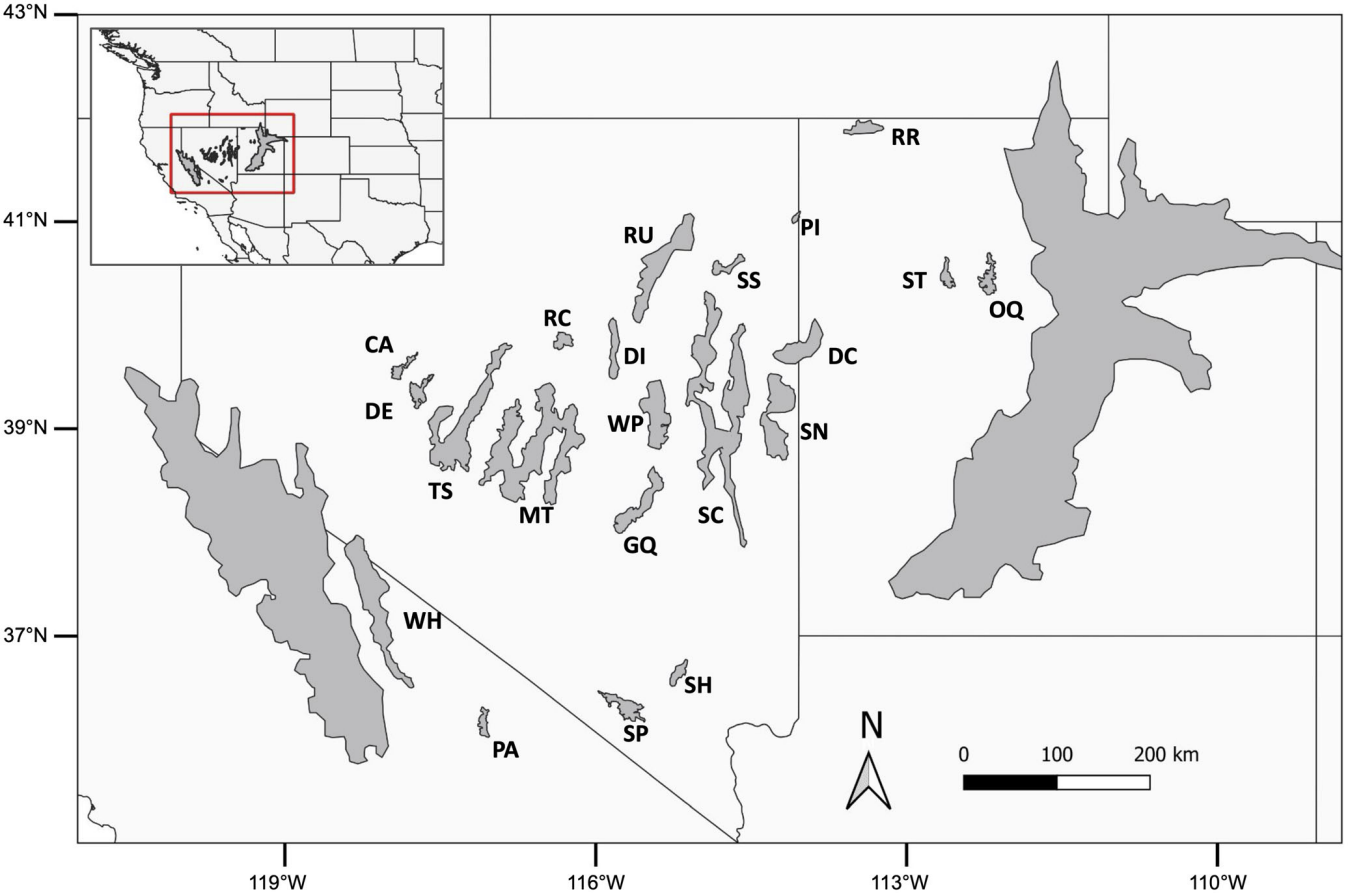
Map of the intermountain region of the western United States, showing the 21 classic Great Basin sky‐islands in Utah, Nevada, and California examined in previous studies, along with the larger mainland sources located to the east (Utah Rockies) and west (Sierra Nevadas). CA, Clan Alpine Range; DC, Deep Creek Range; DE, Desatoya Mountains; DI, Diamond Range; GQ, Grant and Quinn Canyon ranges; MT, Monitor, Toquima and Hot Creek ranges; OQ, Oquirrh Mountains; PA, Panamint Range; PI, Pilot Range; RC, Roberts Creek Range; RR, Raft River Mountains; RU, Ruby Mountains; SC, Schell Creek and Egan ranges; SH, Sheep Range; SN, Snake Range; SP, Spring Range; SS, Spruce and South Pequop Mountains; ST, Stansbury Mountains; TS, Toiyabe and Shoshone ranges; WH, White Mountains; WP, White Pine Range.

To delineate these islands for biogeographic analysis, Brown (1971) used an elevational threshold of 2300 m, which is generally the lower limit of pinyon–juniper woodlands in the Great Basin. However, although necessitated by previous technological limitations, this approach oversimplifies the complexity of the system (Lawlor, 1998). At certain spatial extents, elevation can be useful in discriminating unsuitable and suitable environments for a species because it is correlated with other limiting factors that act on species range limits, such as temperature and precipitation (Hof et al., 2012). However, over larger study regions, elevation alone is not an ideal proxy for suitable sky‐island environments.

In total, 21 Great Basin islands have been classically used for biogeographic analyses in this region (hereafter referred to as “classic islands”). This includes all peaks that are above the aforementioned elevational threshold and have received sufficient sampling to produce presence–absence lists that are deemed reliable for the regional pool of small mammals (Kodric‐Brown & Brown, 1993; Lawlor, 1998; Rickart, 2001). For the classic islands, Brown (1971) calculated geometric measurements of island area (hereafter referred to as “G‐area”) and isolation by Euclidean distance to the nearest mainland (hereafter referred to as “G‐isolation”), and these measurements have since been reused in subsequent analyses in this system (Lawlor, 1998; Lomolino & Davis, 1997; Patterson & Brown, 1991). We assembled these measurements from the literature, serving as a comparative benchmark for the methods developed in this paper.

#### Study species

To demonstrate the ecologically informed axes, we selected the Western Water Shrew (*Sorex navigator*). This species is found on both mainland sources and about half of the classic Great Basin islands (*N* = 9), which provides ample spread in presence/absence data for training IDFs. Additionally, *S. navigator* is especially reliant on the mesic conditions characteristic of the sky‐islands. They are semiaquatic habitat specialists, a life history strategy shared by no other species of small mammal in this system (Rickart et al., 2011). Because no other species fill this niche, we can assume that competitive exclusion does not limit the distribution of *S. navigator*, avoiding a factor that often complicates biogeographic interpretations (Anderson et al., 2002; Gutiérrez et al., 2014). This assumption is supported by a previous analysis of species co‐occurrence patterns among Great Basin small mammals (Kohli et al., 2018).

### Ecological niche models

#### Training extent

We limited ENM training data to the mainlands, as the downstream products will be used to predict island occupancy across the Great Basin through the IDF. This avoids issues of circularity in predictions, as well as violation of equilibrium assumptions of ENMs (Anderson, 2013). Specifically, including presences and background points from the islands in the Great Basin may bias the ENM—as the absence of *S. navigator* on an island may be caused by the area and isolation of that island, rather than the climatic environments used by niche models (Anderson & Raza, 2010). To define the training extent, we obtained shapefiles of the Sierra Nevada and Uinta and Wasatch level III ecoregions delineated by the U.S. Environmental Protection Agency (Omernik & Griffith, 2014). We applied a 15 km buffer to these polygons in order to provide ENMs with a variety of matrix conditions in the surrounding lowlands to contrast with the suitable conditions of the mainlands. Fifteen kilometers also serves as a convenient buffer distance as it is the minimum distance between any island and its closest mainland (Brown, 1971; Rickart, 2001).

#### Presence data

To train ENMs, we compiled regional records of *S. navigator* with minimal coordinate uncertainty (<5 km) from 5 museum collections expected to have low rates of misidentification. These high‐quality records are primarily those leveraging georeferencing efforts from the MaNIS project (Mammal Networked Information System; Stein & Wieczorek, 2004), an inter‐museum georeferencing and data integration initiative that revolutionized accessibility to high‐quality mammal specimen information. This query initially yielded 997 records sampled between the years 1905 and 2019. We retained all records within the training extent, but duplicates found within the same raster cell were removed, leading to a total of 178 localities.

#### Background data

Background data are used to inform an ENM on the conditions available across a study region. Random background points across the study region are often used to capture these conditions. However, as typical for heterogeneous biodiversity datasets, the nature of historical mammalian sampling in the region has led to a highly non‐random spatial structure and environmental signal that must be accounted for (Pardi et al., 2020). Here, rather than conducting simple spatial thinning (Aiello‐Lammens et al., 2015) to reduce the effects of such sampling bias, we used an unweighted target‐group background approach (Anderson, 2003; Brizuela‐Torres et al., 2024). This approach assumes that species collected by the same methods will share a similar spatial sampling bias across a region (Phillips et al., 2009); hence, it indirectly but explicitly characterizes these biases. To do this, we used the sampling localities of other small mammals collected with similar trapping methods.

To assemble data for an appropriate target group, we compiled a list of the region's non‐volant small mammal genera from the literature (Brown, 1971; Rickart, 2001; Rowe et al., 2010, 2015). This includes all shrews, rodents, and lagomorphs that are readily documented using standard trapping techniques. Rowe et al. (2010) designed criteria for omitting certain groups of small mammals from community analyses based on standardized trapping methods, which we applied to both the Sierra and the Utah pools of genera. We then obtained records of all members of the selected genera with minimal coordinate uncertainty from the same museum collections as the focal species (*S. navigator*), following the methods for presence data. This query initially yielded 297,472 records sampled between the years 1800 and 2024. We again retained only records within the training extent. Duplicate records found within the same raster cell were removed (the unweighted approach), leading to a total of 2919 background localities.

#### Environmental data

For this example, in a system of stark climatic contrasts, we obtained all 19 bioclimatic variables from WorldClim averaged between 1970 and 2000 at a resolution of 30 arcseconds (Fick & Hijmans, 2017). These variables consist of seasonal and annual aspects of climate derived from monthly averages of precipitation and temperature.

#### Niche modeling

We developed ENMs for *S. navigator* using Maxent (Phillips et al., 2006). Maxent is a machine learning algorithm that contrasts the environmental values of background pixels to those of presences. Following best practices (Elith et al., 2011), we considered a suite of candidate models differing in feature classes and regularization multipliers. Feature classes determine the flexibility of the model's response to an environmental predictor, while higher regularization multipliers increase penalization on model complexity (Anderson & Gonzalez, 2011; Gerstner et al., 2018; Phillips & Dudík, 2008). Here, we considered linear, linear–quadratic, and linear–quadratic–hinge feature class combinations and regularization multipliers ranging from 1 to 5 with a step value of 1. We assessed model performance using corrected Akaike information criterion (AIC_c_) for small samples (Warren & Seifert, 2011). We used the ENMeval package in R (Kass, Meenan, et al., 2021; Kass, Muscarella, et al., 2021) for niche modeling and evaluations.

#### Model transfer

Because the Great Basin itself was not included in ENM training, we transferred the selected model to a larger study region. This transfer was applied over a modified shapefile comprising the Sierra Nevada, Uinta and Wasatch, Central Basin and Range, Mojave Basin and Range, and Northern Basin and Range ecoregions (Omernik & Griffith, 2014). The use of ecoregion boundaries provides a standardized and relatively unbiased delineation of the broader landscape, rather than restricting connectivity analyses to arbitrarily defined political or study‐area limits. This extent was chosen as it includes not only the mainlands and the islands, but also adjacent areas to the north and south that may be important for modeling connectivity.

We analyzed the transferred niche model in two ways: as a continuous map of environmental suitability and as a binary one. For the continuous map, we used the cloglog transformation to yield a predictive scale from 0 to 1. To create a binary map of suitable areas, we applied a threshold that excludes the 10% of training localities with the lowest predicted suitability, in order to produce a more restrictive map that accounts for potential errors or outliers in the occurrence data (Radosavljevic & Anderson, 2014).

### Island delineation

We identified and delineated ecologically informed islands (hereafter referred to as “E‐islands”) within the Great Basin by matching each of the 21 classic islands to patches of suitable environments on the binary map (Figure 3). Due to the topographic and environmental complexities of this system and technological limitations for original island delineation decades ago, patches from the ENM will not perfectly align with the classic islands. We addressed discrepancies in one of two ways, as relevant: (1) When a single, contiguous patch from the ENM extended over multiple classic islands, we treated those islands as one unified E‐island; or (2) If a classic island contained multiple patches from the ENM, we retained the largest one as well as any others that contributed to at least 15% of the total area for that island.

**FIGURE 3:**
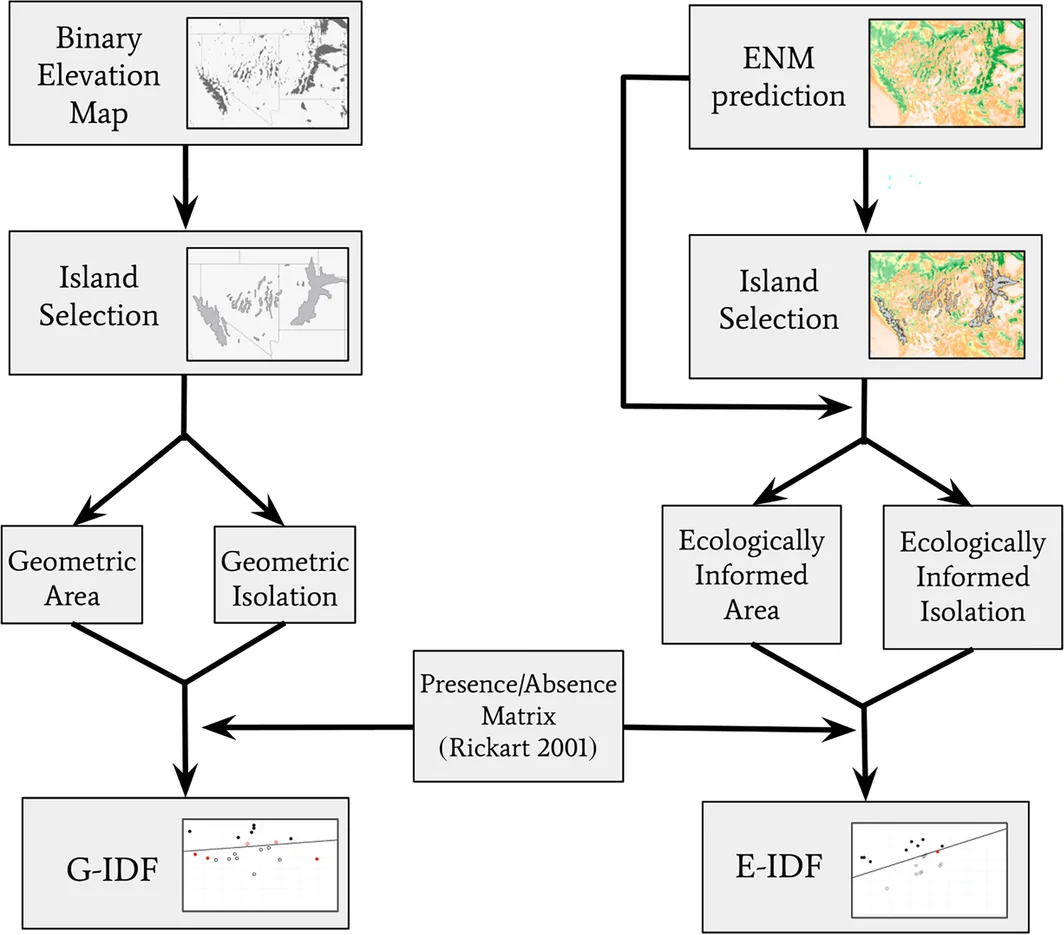
Flowchart comparison of the major steps of two alternative approaches for quantifying area and isolation for insular distribution functions (IDFs). For the geometric‐IDF (G‐IDF; left), classic islands were originally delineated using an elevational threshold. Area and isolation measurements for those are then combined with a presence/absence matrix from Rickart (2001) to generate an IDF. For the Ecologically informed‐IDF (E‐IDF; right), a continuous map of suitability was generated from an ecological niche model (ENM). Islands were then delineated as areas above a suitability threshold and matched to the classic islands in geographic space. The E‐island outlines were then overlaid on the continuous map of suitability to calculate E‐area and E‐isolation. As for the G‐IDF, those measurements for each island were combined with the presence/absence matrix from Rickart (2001) to generate the E‐IDF.

### Ecologically informed measurements

#### E‐area

To derive an ecologically informed quantification of area (hereafter referred to as “E‐area”), we weighted island area by the average suitability value of all pixels found within that island (Figure 4). This yields a metric that simultaneously reflects both the area available for the species and the quality of conditions based on the niche model.

**FIGURE 4:**
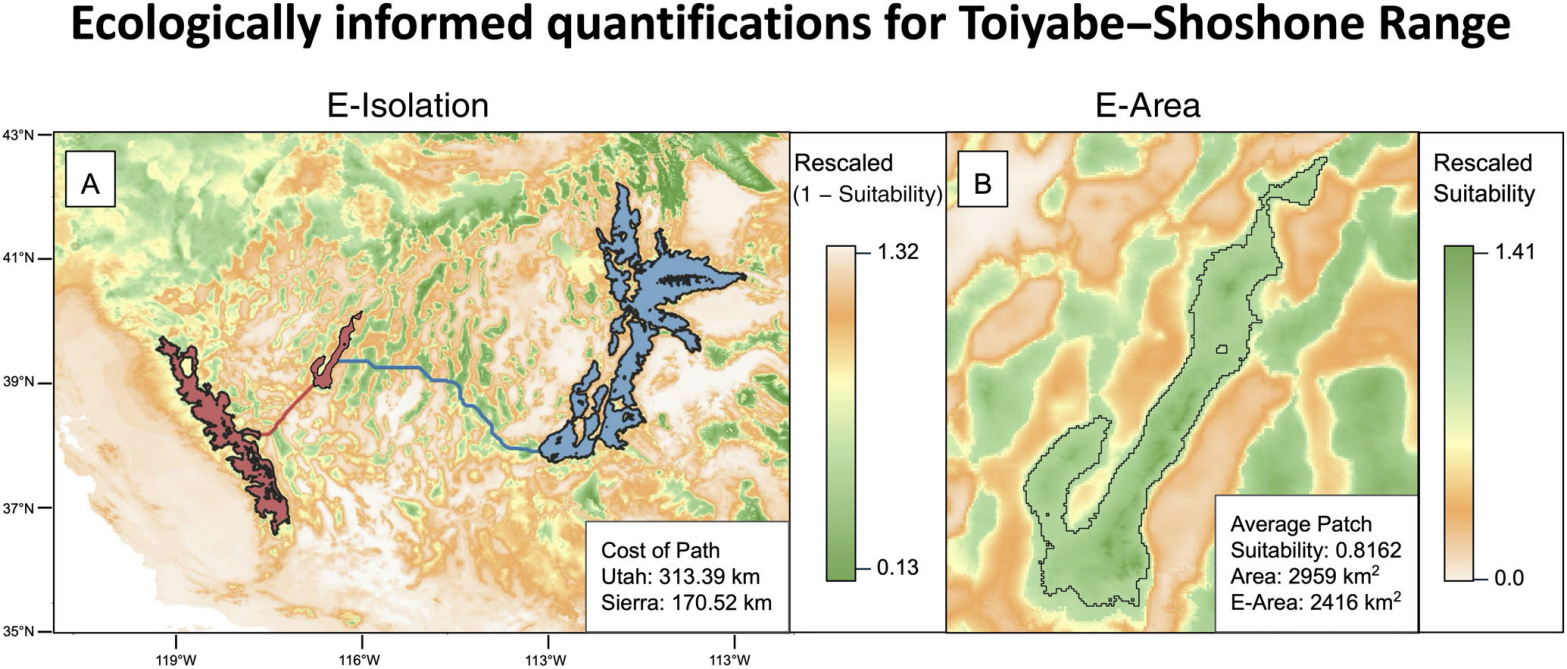
Example of ecologically informed quantification of (A) isolation (E‐Isolation) and (B) area (E‐Area) for the Western Water Shrew (*Sorex navigator*) on a single island, the Toiyabe–Shoshone Range. E‐isolation was calculated as the total accumulated cost along the least‐cost path (after rescaling to preserve units of kilometers), measured separately from the potential sources (Utah Rockies, blue and Sierra Nevadas, red), with the lower value selected. E‐area was calculated by multiplying the island's total area by the average suitability of pixels within the island boundary (after rescaling to preserve units of square kilometers).

Unlike Brown's quantifications, which assume a constant suitability value of 1 for each unit area, the ENM raster includes values of suitability that can vary from 0 (least suitable) to 1 (maximally suitable). To make these area measurements comparable to the classic system (in square kilometers), we adjusted for the heterogeneity in environmental suitability across the mainland and island areas. To do so, we calculated the mean suitability of all mainland pixels and derived a multiplicative scaling factor to set this average to 1. We then applied this scaling factor to all pixels in the suitability raster. This transformation preserves the relative variation in suitability across all pixels while allowing for a valid comparable suitability‐weighted area estimate in square kilometers across the islands.

#### E‐isolation

We calculated ecologically informed quantifications of isolation (hereafter E‐isolation) for E‐islands using least‐cost modeling, which identifies the path of least accumulated resistance between two locations (McRae et al., 2008). We used the continuous map of suitability to infer environmental resistance across the matrix. We inferred resistance as 1 − suitability (Duflot et al., 2018; Kupfer, 2012), creating a transition layer to measure conductance between pairs of pixels (Resasco & Fletcher, 2021; Van Etten, 2017). For each E‐island, we calculated an LCP between that island's centroid and the edge of either mainland. We considered the cheapest path of the two to be that island's E‐isolation (Figure 4). We used the gDistance R package (Van Etten, 2017) for least‐cost modeling.

The classic system assumes a uniform resistance across the entire matrix, making isolation cost directly proportional to Euclidean distance. In the heterogeneous matrix, however, resistance values vary based on environmental suitability (as noted above for area), resulting in a lower average resistance across the matrix. To achieve comparability of units with the classic system (in kilometers), we rescaled the heterogeneous matrix of resistance values before measuring costs. To do so, we calculated the mean resistance across the matrix and derived a multiplicative scaling factor, which we then applied to all resistance values. This rescaling preserves the heterogeneity of the matrix while ensuring that on average resistance values match the constant resistance assumed in the classic system. We then used this rescaled raster to calculate the accumulated cost along the LCP, allowing for a valid comparison of isolation (in kilometers).

### Insular distribution functions

Using presence–absence data from Rickart (2001), we developed two IDFs (Figure 3). The first IDF (hereafter referred to as “G‐IDF”) was fitted using G‐area and G‐isolation measurements, as done by previous authors in various systems (Lawlor, 1998; Lomolino & Davis, 1997). The second IDF (hereafter referred to as “E‐IDF”) was fitted using E‐area and E‐isolation. In both cases, we log‐transformed area measurements as done by previous authors (Frick et al., 2008; Lawlor, 1998; Lomolino, 1986; Lomolino & Davis, 1997). IDFs built with arithmetic (untransformed) values of area are expected to follow a curvilinear pattern, as isolation will eventually exceed the maximum dispersal capacity of a species even as islands continually get larger (Lomolino, 1986). Therefore, a semilog plot better reflects the expected biological relationship while also linearizing the resulting IDF for clearer interpretability.

We fitted IDFs using linear discriminant analyses (LDA; Fisher, 1936), a common multivariate classification technique. We opted not to use logistic regression, which typically requires at least 20 data points per variable (Efron, 1975), meaning that at least 40 islands would be necessary for this two‐dimensional analysis (Lomolino et al., 1995). We compared the performance of IDFs using binary classification errors as well as inspection of their eigenvalues, which characterize the discriminatory power of the generated functions (degree of class separation). We used the klaR (Roever et al., 2023) and MASS (Ripley et al., 2025) R packages for performing LDA.

### Simulations of data degradation

While the Great Basin has received enough sampling effort to produce reliable presence–absence lists for the islands included in this empirical analysis (Rickart, 2001), potential analyses in other systems will likely contain higher island‐level false‐absence rates due especially to inadequate sampling (such that a species may appear absent from an island where it really does occur). In contrast, although false‐presence errors indeed can occur (e.g., due to species misidentifications or incorrect georeferences that erroneously assign a record to a given island), overall, they should be less common than false absences. To determine the effects of the potential data bias caused by inadequate sampling, we conducted a series of simulations. The simulations assessed differences in slope and intercept at different levels of data degradation (bias). Specifically, we aimed to determine whether the differences between the ecologically informed insular distribution function (E‐IDF) and geometric IDF (G‐IDF) in the original analysis persisted when true presences are sometimes undetected.

We considered three levels of detection probability that introduce bias into the island occupancy data of Rickart (2001), mimicking situations more likely to be encountered without intensive focused sampling. The chosen detection probabilities (*p* = 0.95, 0.90, 0.85) correspond to island‐level non‐detection probabilities (or false absence rates) of 0.05, 0.10, and 0.15, respectively. For this exploration of the effects of non‐detection on observed patterns and biological inferences, we chose these levels to introduce errors corresponding to moderate but not extreme sampling inadequacy. For each detection probability, we generated 200 replicate datasets by converting each true presence to a false absence with probability 1 − *p*, conditional on the species being truly present on an island. True absences remained unchanged, and no false presences were introduced. Thus, the simulations represent a simplified detection process in which all occupied islands share the same probability of a presence being recorded. For each simulated presence–absence dataset, we refit both the G‐IDF and E‐IDF as described above and extracted the resulting slopes, intercepts, classification error rates, and eigenvalues.

## RESULTS

### Niche models

Of the 15 candidate models, the one selected as optimal used linear, quadratic, and hinge feature classes and a regularization multiplier of 2. Those settings corresponded to high flexibility for the modeled response and higher protection against overfitting than the default value. In addition to having the lowest AIC_c_ value, it also ranked among the highest values of performance under cross‐validation (validation area under the reciever operating characteristic curve = 0.696, 10% validation omission rate = 0.126). When applied to the study region (Appendix S1: Figure S1), the model showed strong discrimination between matrix and island environments. In addition to the mainlands and the islands, the model prediction also shows substantial areas of suitable environments just north of the Basin.

### Island delimitation

From the binary map of suitable environments (Appendix S1: Figure S1), most of the classic islands (as originally delineated by elevational cutoffs) matched clearly with distinct patches from the ENM. This led to 19 E‐islands in total, as two classic islands were no longer independent insular units based on the ENM. The Raft River Range (RR) became contiguous with the larger suitable areas to the north of the Basin, so we omitted it from the list of islands. Additionally, the Diamond Range (DI) became contiguous with the Monitor Range (MT). We treated these as a single E‐island and accounted for this in the classic island dataset to maintain equivalency. For the classic data, we summed the areas of MT and DI and used the minimum isolation of the two. Additionally, the Schell Creek Range (SC) consisted of multiple substantially sized insular patches, which we treated as a single island.

Comparing G‐quantifications to E‐quantifications revealed differing patterns in regards to changes to island area and isolation (Appendix S1: Figure S2). Overall, quantifications of the majority of islands increased from G‐area to E‐area, although these increases generally did not diverge dramatically from a 1:1 expectation. In contrast, G‐isolation and E‐isolation scaled tightly in a linear manner across most of the dataset, with the exception of the most isolated islands (Appendix S1: Figure S2).

### Insular distribution functions

As expected, the E‐IDF outperformed the G‐IDF (Figure 5). It made four fewer classification errors (error rate: 0.053) compared with the G‐IDF (error rate: 0.263). Furthermore, the E‐IDF produced a discriminant function with stronger class separation, as indicated by a higher eigenvalue from the LDA (E‐IDF eigenvalue: 44.81; G‐IDF eigenvalue: 7.98). Eigenvalues reflect the function's ability to differentiate groups relative to within‐group variance, with larger values indicating stronger separation.

**FIGURE 5:**
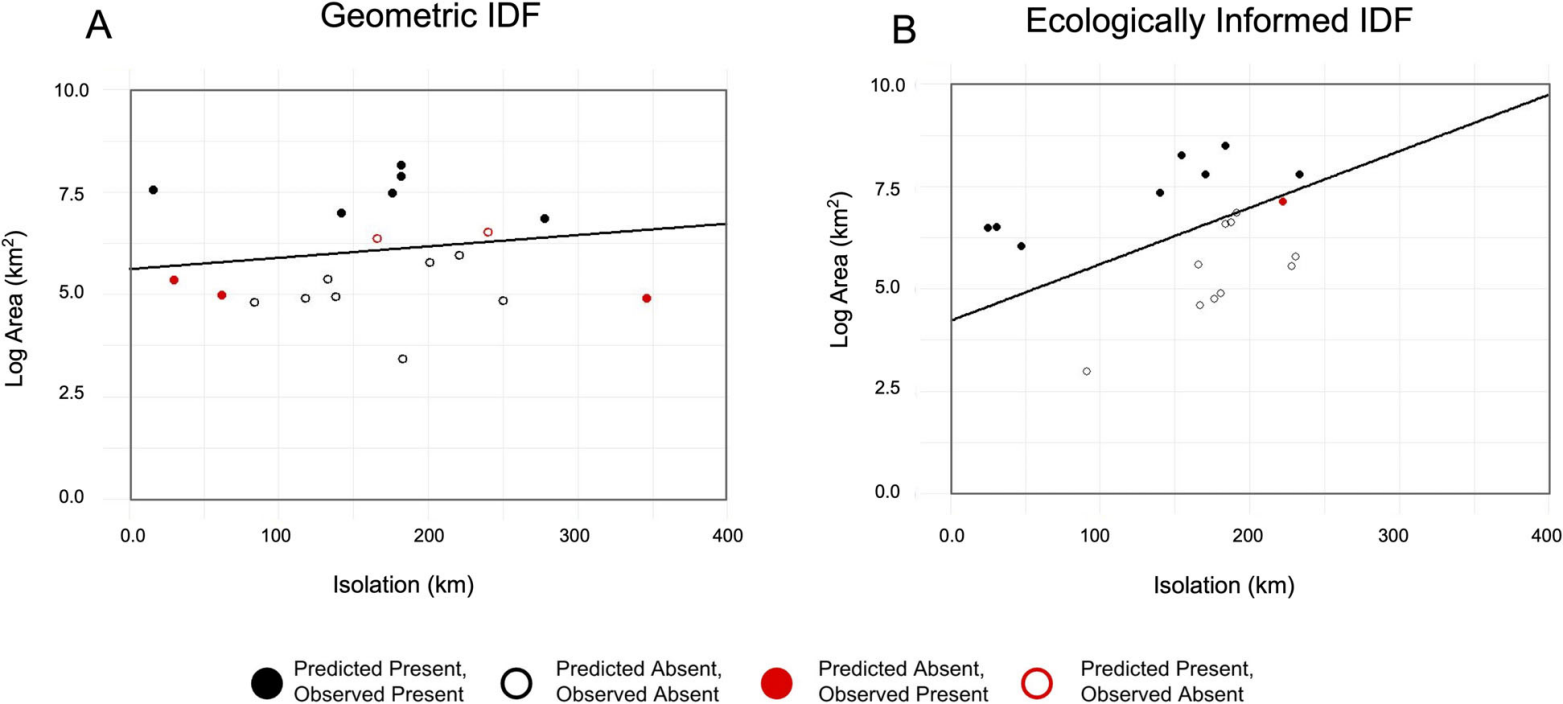
Comparison of insular distribution functions (IDFs) for the Western Water Shrew (*Sorex navigator*) fitted with (A) geometric quantifications of area and isolation (Geometric IDF) and (B) ecologically informed quantifications of area and isolation (Ecologically Informed IDF).

Importantly, the two IDFs led to different inferences regarding the biogeographic factors limiting the insular distribution of *S. navigator*. These inferences rely on comparison of the slopes and intercepts of the IDFs (Figure 1), where higher slopes reflect a greater effect of isolation and higher intercepts suggest a stronger effect of area. Both IDFs yielded patterns that would be described as compensatory (Figure 1D), because inferred area requirements increased as isolation increased. Nevertheless, the E‐IDF had a much steeper slope (0.0138) compared with the G‐IDF (0.00278; Figure 5). Additionally, the E‐IDF showed a lower intercept (4.12) than the G‐IDF (5.49). Because the models are plotted in semilog space, this 25.0% difference in intercepts corresponds to a 74.6% reduction in untransformed units.

### Simulations

Conclusions from the empirical analysis using the presence–absence data from Rickart (2001) were largely consistent with findings using the simulated (data‐degraded) datasets. For both E‐IDF and G‐IDF models, increasing the probability of false absences on given islands led to greater variability in slope and intercept estimates (Appendix S1: Figures S4–S9). Additionally, they showed slight tendencies toward estimates of higher slopes and lower intercepts. Within each replicate (maintaining the same degraded data), data error caused shifts for both models in the same direction, so that changes in slope and intercept occurred in concert between the G‐IDF and E‐IDF (Appendix S1: Figures S10 and S11). Despite this increasing variability in slopes and intercepts as detection probability decreased (i.e., with more false‐absences), E‐IDFs still produced steeper slopes and lower intercepts than G‐IDFs fit to the same degraded datasets across all simulations (Appendix S1: Figures S10 and S11).

Overall, incorporating ecologically informed measures of area and isolation continued to substantially alter inference relative to the classic geometric framework, even with the simulations inserting data bias. Critically, the core differences between the two frameworks persisted, with the E‐IDF exhibiting a steeper slope and lower intercept than the G‐IDF. In addition to indicating higher performance, this reiterates a greater role for isolation and a reduced minimum‐area threshold for persistence. Hence, the biological conclusions were robust to moderate data bias regarding false absences.

## DISCUSSION

The methods and analyses employed here expand upon Lomolino's (1986) hypotheses about insular distributions, bringing patterns based in a binary conceptualization of the world into a continuous one. By uniting a classical approach with subsequent advancements in theory and methods, we accounted for key insufficiencies of the original IDF framework, leading to a model with stronger predictive power and yielding novel biological conclusions. At least for this study species, the results indicate that extinction and colonization dynamics in the Great Basin were more structured by environmental suitability and landscape permeability than previously recognized, with a greater relative role for immigration versus extinction. These insights set the stage for a deeper exploration of how ENM‐based metrics improve our understanding of island and patch occupancy patterns—and the processes that influence them—in this system and beyond.

### Model performance and biological conclusions

The E‐IDFs counter earlier interpretations of this system. IDFs have been applied to mammals of the Great Basin multiple times by previous authors (Lawlor, 1998; Lomolino, 1986; Lomolino & Davis, 1997), all using the geometric quantifications developed by Brown (1971). A central takeaway of those studies was that most of the Great Basin mammals analyzed (including *S. navigator*) exhibited IDFs with flatter slopes compared with the faunas of other examined archipelagos, such as non‐volant mammals inhabiting islands of the Thousand Islands region of southeastern Canada and northeastern United States (Lomolino, 1986) and the sky‐islands of the Cordillera‐Madrean region of the southwestern US and northwestern Mexico (Lomolino & Davis, 1997). Those results align with Brown's original hypothesis of non‐equilibrium island biogeography: a paradigm specifically dominated by faunal relaxation for sky‐island mammals in the Great Basin driven only by extinction (Brown, 1971). During the last glacial maximum (LGM; ~20,000 years ago), cooler, wetter climates would have increased mesic conditions across the low‐elevation matrix, allowing differential colonization where most species established populations on some or all of the islands. Subsequent Holocene warming and drying then imposed differential extinction pressures on the insular populations that had colonized islands, contributing to present‐day patterns of distribution. An IDF slope near zero thus supports the solely relaxation hypothesis, suggesting that while the examined species could reach every island, long‐term persistence occurred only on islands remaining above a critical minimum area.

In contrast, the current results from the E‐axes depart from the relaxation hypothesis. The substantially steeper slope seen in the E‐IDF reflects a more pronounced trend of increase in minimum log‐area requirements with greater isolation, with the value for slope effectively serving as an inverse measure of inferred colonization propensity (Lomolino, 1999; Peltonen & Hanski, 1991). Under this interpretation, *S. navigator* would be far more isolation‐limited than previously suggested: Even islands large enough to support populations may be unoccupied if immigration rates were not sufficiently strong. At the same time, the substantially lower intercept of the E‐IDF indicates a reduced minimum‐area requirement under conditions where colonization was not limiting for the islands of high connectivity. This pattern is not consistent with a distribution of this species solely relaxation‐dominated (i.e., only differential extinction). Instead, it suggests the additional influence of potential processes such as differential colonization at the LGM (of some but not all islands), post‐Pleistocene immigration to some islands, or even incomplete relaxation for less isolated islands where populations on some small islands have undergone reductions but not yet been extirpated (Lomolino & Davis, 1997). This change in biological interpretation persisted across data‐degraded simulations as well, where introducing false absences into the island occupancy data showed slight directional trends regarding slope and intercept estimates (and much greater variability for them), but the relative differences in slopes and intercepts between E‐IDFs and G‐IDFs remained clear and consistent.

These differences arise from how environmental heterogeneity reshapes both E‐area and E‐isolation. For example, regarding area, the two islands exhibiting the largest departures from the 1:1 expectation between G‐area and E‐area—Panamint Range (PA) and Pilot Range (PI)—were also among the smallest islands by G‐area. Yet, these islands differ markedly in climatic conditions at higher elevations. PA, situated on the western edge of the Basin, is considerably hotter and drier, whereas PI lies in the cooler, wetter north. Their E‐areas diverged accordingly, illustrating how continuous suitability metrics can amplify or reduce areal estimates in ways that geometric boundaries cannot capture.

Similarly, the E‐isolation characterizations captured two distinct phenomena not accounted for when using G‐isolation. First, the LCP emulated potential “stepping stone” routes that could have facilitated colonization (Brown & Kodric‐Brown, 1977). Frequently, the optimal path to one island traversed another island along the way (Appendix S1: Figure S3). This is because suitability values on island pixels are high enough that crossing them incurs minimal costs. This contrasts with G‐isolation, where distances between the mainland and an island are fixed regardless of whether the Euclidean path crosses over an island or through harsher matrix conditions. Consequently, the LCP reduces perceived isolation for interior islands in the Basin (Appendix S1: Figure S2). Second, LCP analyses revealed the alternative paths for islands closest to the Utah source. Unlike the paths to the Sierras, which were more direct across the matrix, the paths to the Utah Rockies either came from the south and took advantage of stepping stones, or came from the north and leveraged suitable environments north of the Basin (Appendix S1: Figure S3). In fact, this northern region may have supported post‐Pleistocene immigrations for some more northerly islands into the present.

### Methodological extensions

Several methodological choices made in this study highlight a variety of possible future extensions. First, alternative resistance‐based methods beyond LCP analyses can be implemented for calculating isolation. For example, random walks (Codling et al., 2008; Resasco & Fletcher, 2021) and kernel densities (Compton et al., 2007; Long et al., 2021) could be used for calculating isolation of an island as a probability based either on many paths or a radius of dispersal. Here, we employed LCP analysis, which was most analogous to the classic metric of Euclidean distance and therefore best appropriate for this comparative study. More broadly, the specific environmental variables used to quantify matrix permeability will depend on system and scale. In the present study, we worked at the extent of an ecoregion characterized by strong climatic gradients, making climate‐based predictors a natural choice for estimating suitability and resistance. In other systems, particularly at smaller spatial extents and finer grains (e.g., forest–agriculture mosaics), land cover or remotely sensed habitat structure may provide more appropriate inputs. Importantly, however, the principle remains unchanged: Isolation should be quantified as resistance to movement through a heterogeneous matrix rather than as uniform geometric distance.

Second, another consideration to build upon is the choice of function used for expressing the form of the IDF, which also points toward use of ecologically informed quantifications for other analyses that link population and landscape ecology. Here, we implemented linear functions, the simplest for drawing biological conclusions and the most direct comparison with the traditional G‐IDFs. Incorporating quadratic or sigmoidal functions may be relevant for fitting IDFs for species with more complex factors beyond just extinction and colonization dictating their distribution, such as competitive exclusion (Lomolino, 1999). Additionally, machine learning methods such as support vector machines (Drake et al., 2006) or neural networks (Deneu et al., 2021) could add predictive power to these models. More importantly, the ecologically informed area and isolation metrics developed here are not specific to just the IDF framework. The same predictors could be incorporated into logistic regression, hierarchical occupancy models, or other approaches for modeling species occupancy (MacKenzie et al., 2017). Such flexibility is particularly relevant when imperfect detection must be accounted for explicitly (Guillera‐Arroita, 2017). However, regardless of the modeling framework employed, island systems will almost always provide only low sample sizes for model training; therefore, the number of predictive features used should be limited (Lomolino et al., 1995) and the complexity of the machine learning algorithm should be controlled (Kass, Meenan, et al., 2021; Kass, Muscarella, et al., 2021).

### Future directions

The heuristic potential of the single‐species IDF model broadens greatly when applied to multiple species. Lomolino initially described the IDF as hierarchical, capable of addressing patterns at levels ranging from a single species to entire communities and even to comparisons across archipelagos (Lomolino, 1999). This study presents a single‐species implementation to establish a clear proof of concept, demonstrating how refined quantifications influence inference, setting the stage for later studies to introduce additional complexity associated with multispecies analyses. Further expanding the current analyses to other Great Basin mammals could identify differences among them in the roles of extinction and colonization tradeoffs. Any differences in the slopes and intercepts of IDFs could then be examined for associations with functional traits related to colonization and persistence (Kohli et al., 2021; Kohli & Rowe, 2019). Additionally, superimposing IDF predictions for multiple species within the same regional pool generates community‐level predictions of occupancy across islands (Lomolino, 1986). More generally, the ecologically informed predictors developed here also could be incorporated into more complex metacommunity or joint species distribution modeling frameworks (Guisan & Rahbek, 2011; Ovaskainen & Soininen, 2011), where additional biological processes are modeled explicitly.

The application of E‐IDFs would gain further value with the incorporation of past environmental conditions. A core assumption of IDFs, and most models used in metapopulation and metacommunity ecology (Howell et al., 2018; Leibold et al., 2004; Thuiller et al., 2013), is that a static environment with constant patch area and isolation (typically in the present) adequately predicts occupancy by reflecting the effects of extinction and colonization (implicitly or explicitly over ecological time; Lomolino, 1999). This assumption does not consider the effects of hysteresis in insular systems, where present outcomes depend not only on the current system state but also on past conditions. The Constraint‐based model of Dynamic Island Biogeography (C‐DIB) invokes hysteresis and conceptually addresses this gap, providing a framework to predict insular community composition in *environmentally dynamic* systems by incorporating past environmental conditions and species occupancy patterns along with species traits (Burger et al., 2019). To facilitate implementations of IDFs under the principles of the C‐DIB, recent improvements in climatic reconstructions from global circulation models (Lima‐Ribeiro et al., 2015) enable the transfer of ENMs to past conditions (Guevara et al., 2018) and allow estimation of prior patch area and isolation. For example, because most colonizations in the Great Basin presumably would have occurred at the LGM, past isolation could be measured as the LCP between a present patch and either mainland using a suitability map at that time. This explicit inclusion of past information could improve the predictive power of IDFs as well as further our understanding of how species and communities responded to events of environmental change.

Integrating data‐driven models like IDFs into conservation planning holds promise, especially for improving species threat assessments and identifying at‐risk subpopulations in data‐limited systems. For example, the International Union for the Conservation of Nature (IUCN) requires detailed species distribution information for threat assessments, which can be supported by ENMs and characterizations of sampling effort and population presence (Anderson, 2023). Although broad‐stroke distribution metrics, such as area of occupancy (AOO) and extent of occupancy (EOO), form the basis of most risk assessments, understanding fragmentation is crucial for evaluating subpopulation risk (IUCN Standards and Petitions Committee, 2024). A taxon is considered “severely fragmented” when it faces a higher extinction risk because most individuals are confined to small, isolated subpopulations, which may go extinct with limited chances for recolonization (IUCN Standards and Petitions Committee, 2024). IDFs can help determine extinction risk and go beyond it, characterizing subpopulation vulnerability by capturing the interaction between extinction and colonization for subpopulations. Populations that are predicted to be extinct but are in fact still persisting may indicate extinction debt (Kuussaari et al., 2009) and risk of eventual future extirpation.

Extending E‐IDFs to other systems of course requires reliable presence and absence data for a sufficient number of patches. In the Great Basin sky‐island system of small non‐volant mammals, the E‐IDF only included presence–absence data for the islands previously analyzed by other researchers; many suitable patches from the ENM (but considered insufficiently surveyed) were excluded from fitting the model (Figure 6). These omitted patches represent precisely the kind of patches with unknown occupancy status for which IDFs may be most useful. Placing them in biogeographic space and applying the fitted IDF would generate predictions of occupancy and identify islands for future sampling (those suggested as likely occupied). Addressing this information gap would benefit from mobilizing data from smaller regional museums and herbaria that currently lack digitized and georeferenced records, much needed generally in biodiversity informatics (Anderson, 2012; Kissling et al., 2018). Importantly, the simulations conducted here incorporating false absences suggest that biological conclusions will remain robust to moderate levels of sampling bias, supporting the application of this framework in such systems.

**FIGURE 6:**
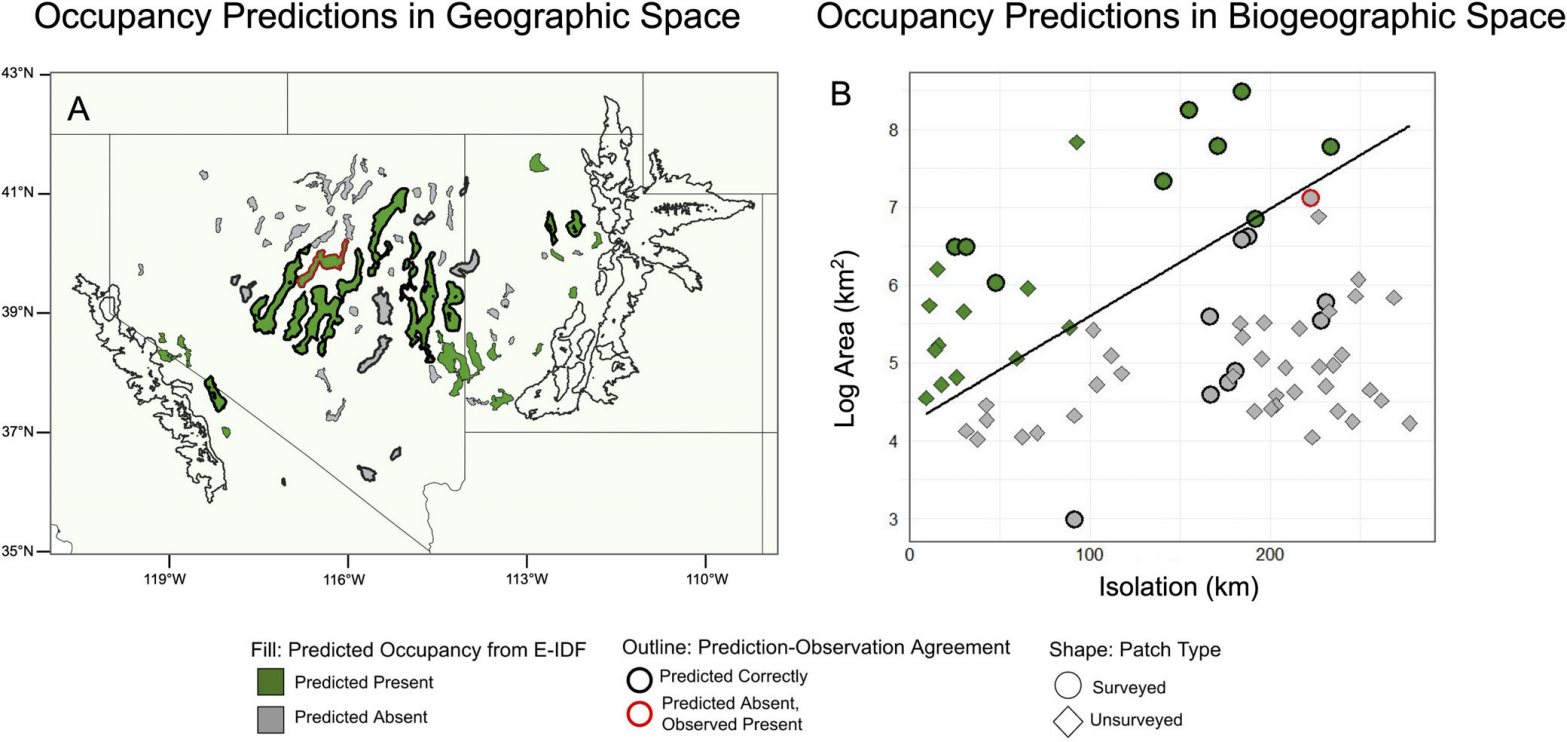
Predicted and observed occupancy for the Western Water Shrew (*Sorex navigator*) on the 19 classic Great Basin islands and predicted occupancy of unsurveyed patches (the 50 largest unsurveyed patches as delineated by the ecological niche model). (A) Map of surveyed and unsurveyed patches colored by predicted occupancy based on the ecologically informed insular distribution function (E‐IDF). Green fill indicates patches predicted present; gray fill indicates patches predicted absent. Bolded outlines indicate that a patch has been surveyed, with black outlines indicating agreement with prediction and red ones denoting disagreement. (B) Surveyed and unsurveyed patches plotted in biogeographic space and E‐IDF indicating the discriminant function that modeled presence or absence. The line denotes the fitted decision threshold from the IDF. Circles represent surveyed patches, and diamonds represent unsurveyed ones. Fill and outline follow the same conventions as for panel (A).

In closing, this study builds on Lomolino's IDF framework, transforming it from a binary to a continuous characterization, as well as enhancing its predictive accuracy, ecological realism, and practical relevance. These results indicate that present‐day environmental heterogeneity has strong explanatory power regarding current occupancy patterns for this species in the Great Basin, setting the stage for investigating the role of past environmental change and expansion to community‐level analyses. This refined approach to quantifying area and isolation for biogeographic modeling holds relevance to related analyses in population and landscape ecology and can also enhance biodiversity assessments and decision‐making.

## CONFLICT OF INTEREST STATEMENT

The authors declare no conflicts of interest.

## Supporting information


Appendix S1.

## Data Availability

Data and code (Gaier, 2026) are available in Figshare at https://doi.org/10.6084/m9.figshare.29564732.v1.
