# Supplementary material for: Improving island occupancy predictions using ecologically informed metrics of area and isolation
Source: Ecology. 2026 Oct 21;107(10):e70541. doi: 10.1002/ecy.70541 (PMC13686798; doi:10.1002/ecy.70541)

Appendix S1

Improving island occupancy predictions using ecologically informed metrics of area and isolation

Andrew G. Gaier, Brooks A. Kohli, Robert P. Anderson

Ecology

Citation in article	Description
Figure S1	First figure in Appendix S1
Figure S2	Second figure in Appendix S1
Figure S3	Third figure in Appendix S1
Figures S4-S11	Figures from simulation analysis

Figure S1

Island delimitation process. The selected Maxent model was applied to the study region to map continuous environmental suitability in the Great Basin for *S. navigator*. A threshold was applied to the continuous suitability map to create a binary map of suitable conditions. From this binary map, only suitable patches that aligned with classic islands to define the new island boundaries were retained.

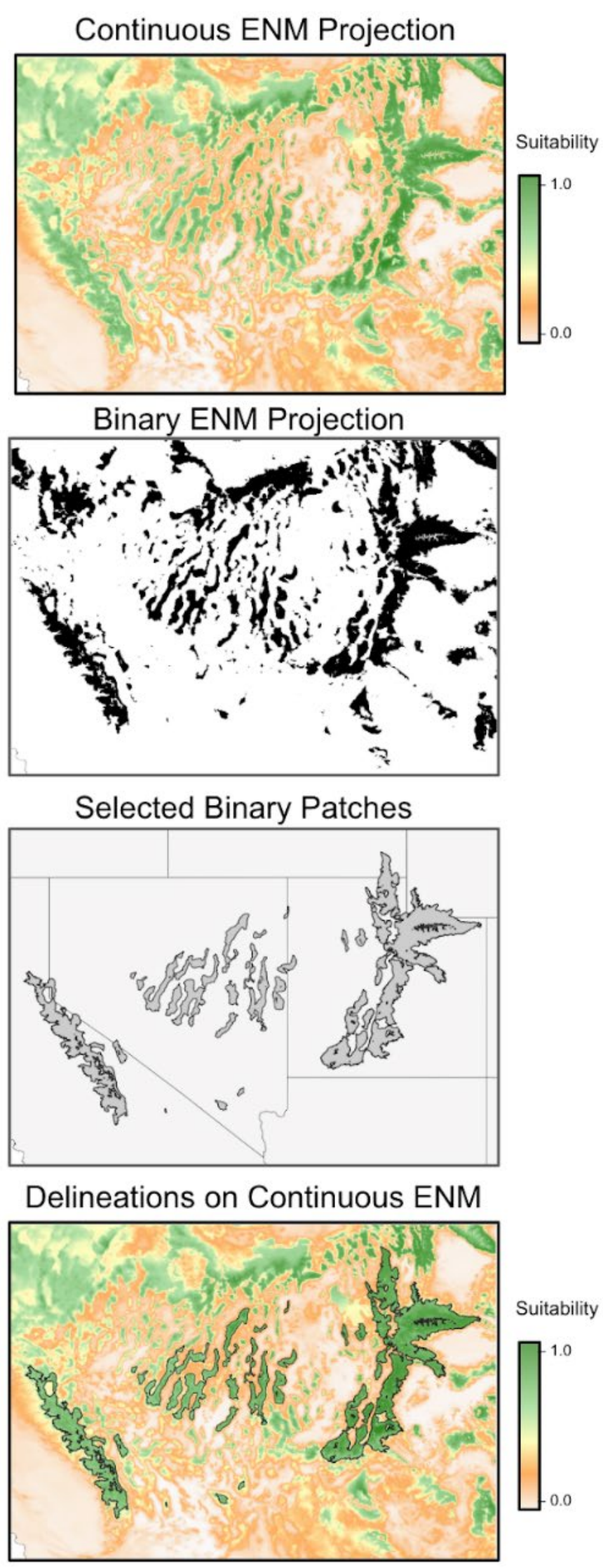

Figure S2

Changes in island characteristics for *S. navigator* in the Great Basin, showing plots of E-isolation against G-isolation (left) and E-area against G-area (right). Dashed line represents a 1:1 ratio between the different quantifications. Fill color indicates nearest mainland source of islands determined from least-cost path analyses.

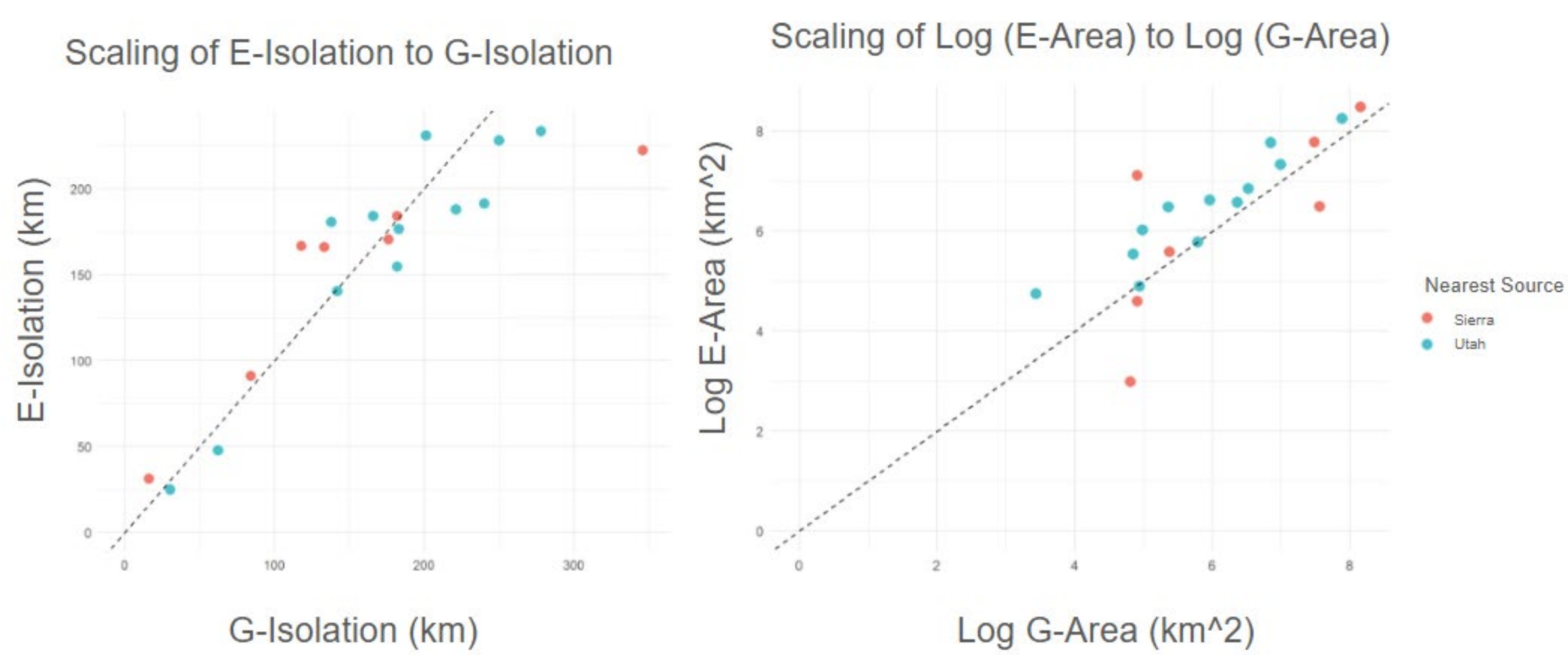

Figure S3

Selected least-cost paths (CPs) from mainland source regions to each Great Basin island for *S. navigator*. For every island, CPs were calculated across the resistance surface separately from the Sierra Nevada and Utah Rocky Mountain mainlands. The path with the lower cost was selected and assigned to that island. Path color denotes the inferred mainland source: red for Sierra-derived paths; blue for Utah-derived paths. Islands with confirmed *Sorex navigator* presence are marked with hatching.

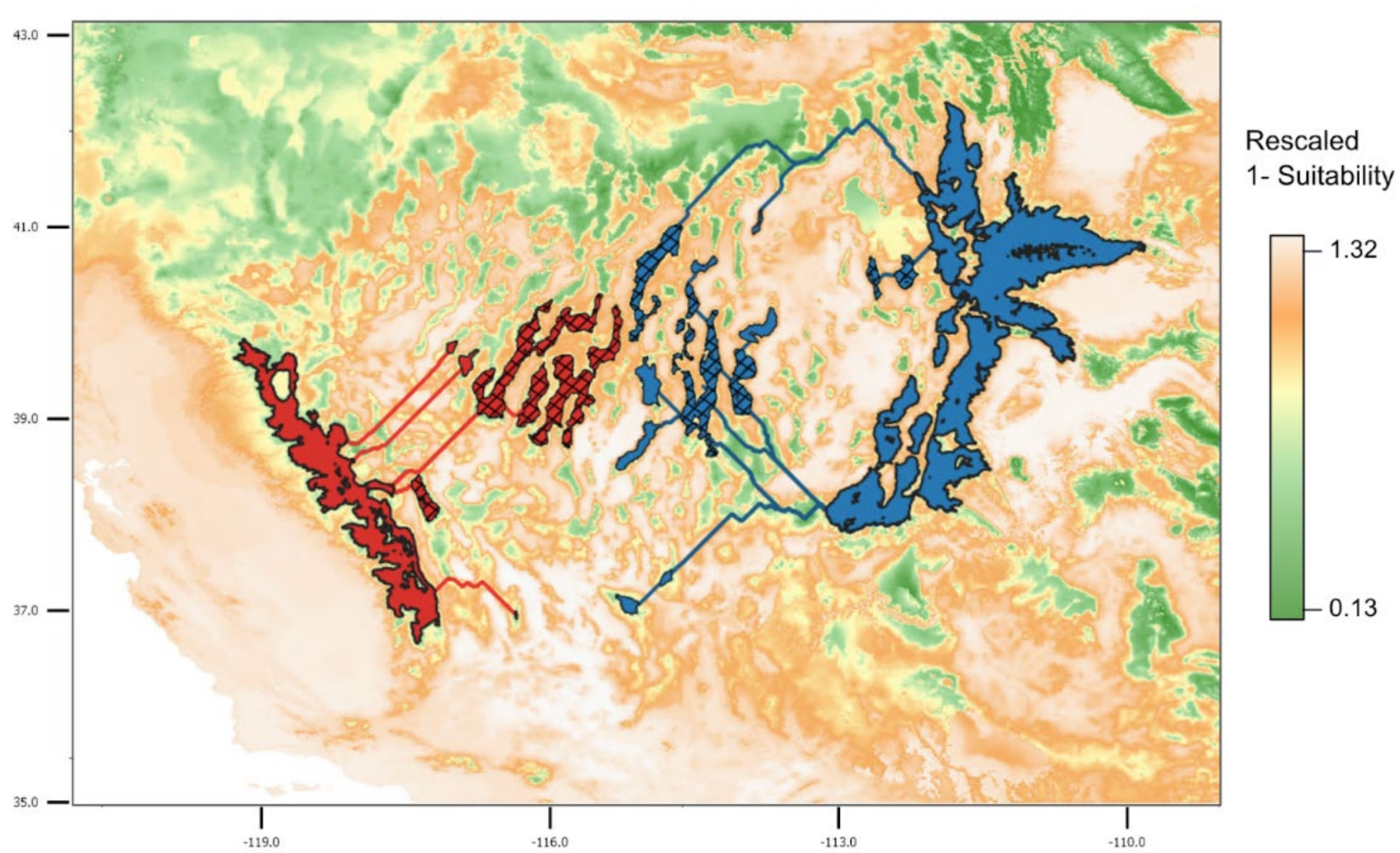

Figure S4

Variation in ecologically informed Insular Distribution Functions (E-IDFs) under different simulated levels of detection probability. Shaded bands represent the spread of simulated IDF boundaries diverging from the baseline (black line). Inner, mid, and outer bands represent the interquartile range (25–75%), the 5–95% range, and the full range (minimum to maximum) across all simulations.

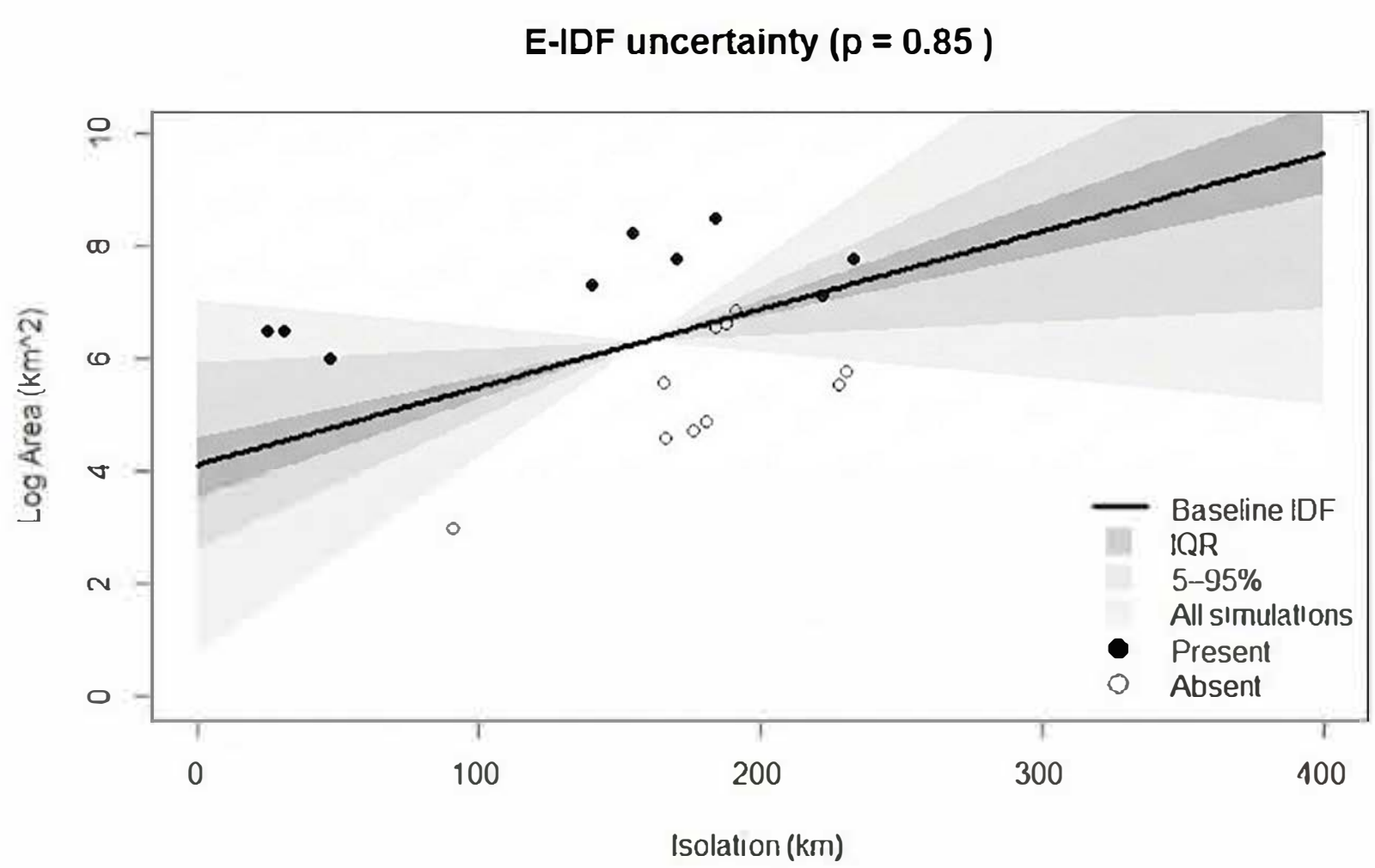

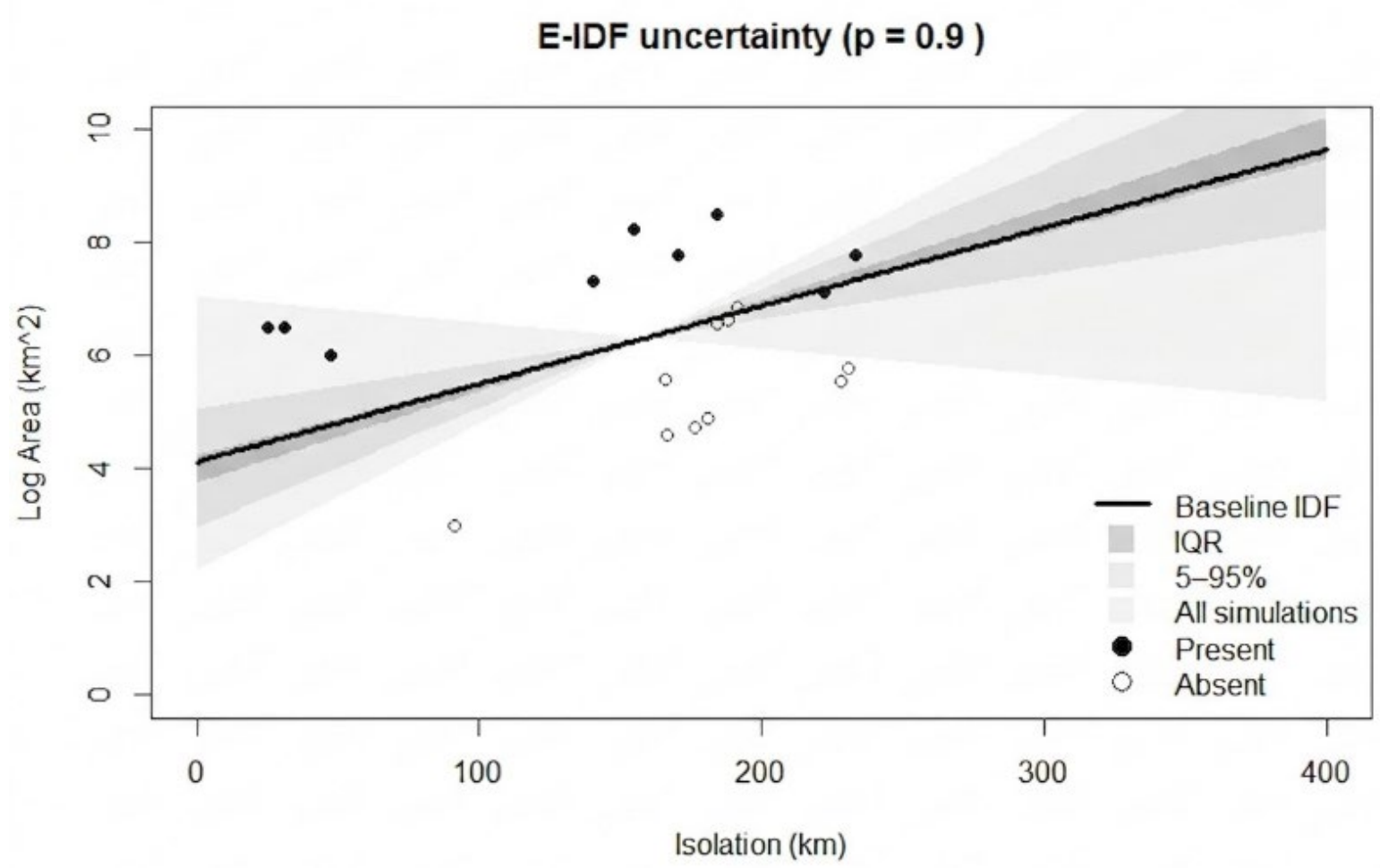

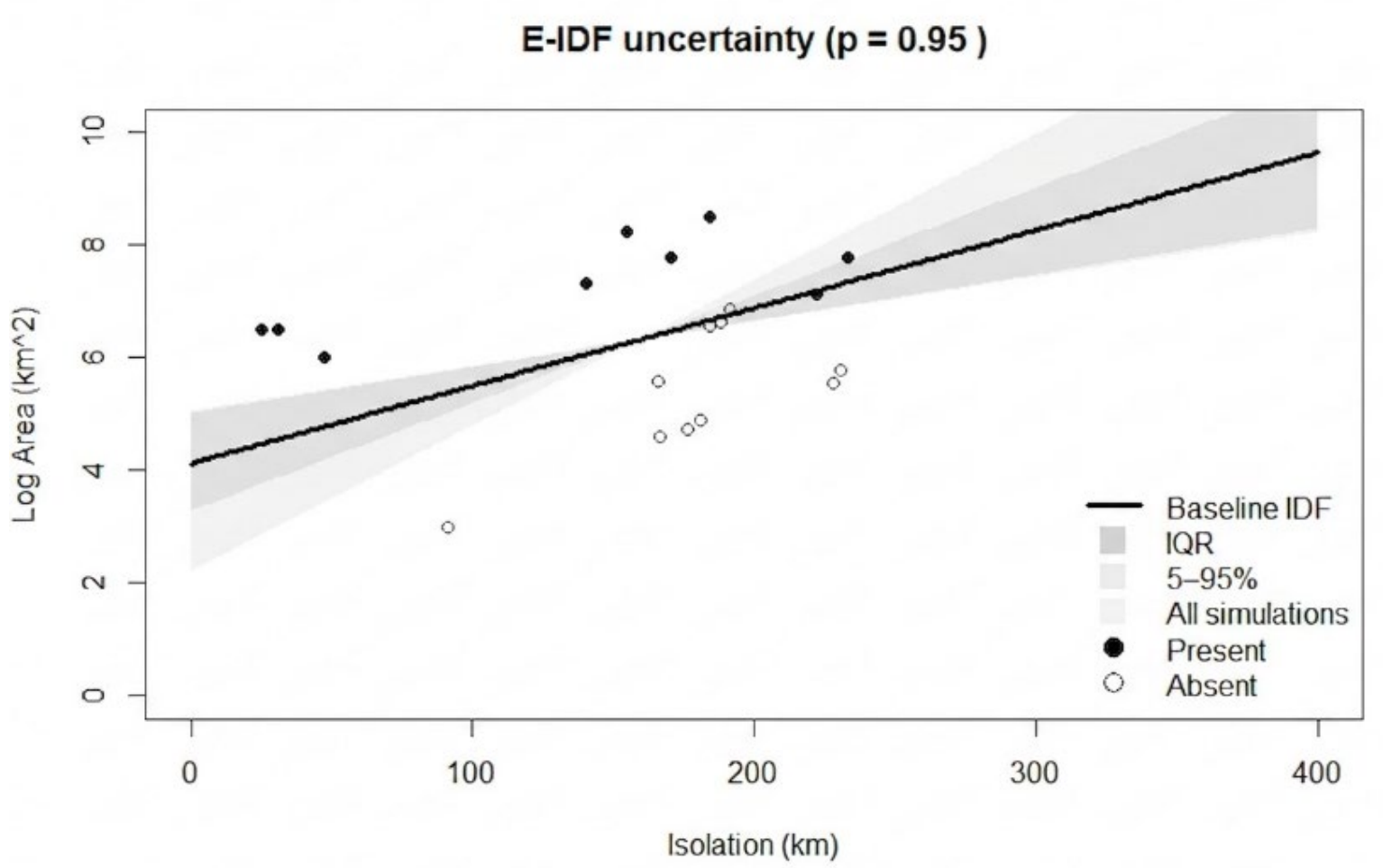

Figure S4 (continued from previous page)

Figure S5 (following page)

Variation in geometric Insular Distribution Functions (G-IDFs) under different simulated levels of detection probability. Shaded bands represent the spread of simulated IDF boundaries diverging from the baseline (black line). Inner, mid, and outer bands represent the interquartile range (25–75%), the 5–95% range, and the full range (minimum to maximum) across all simulations.

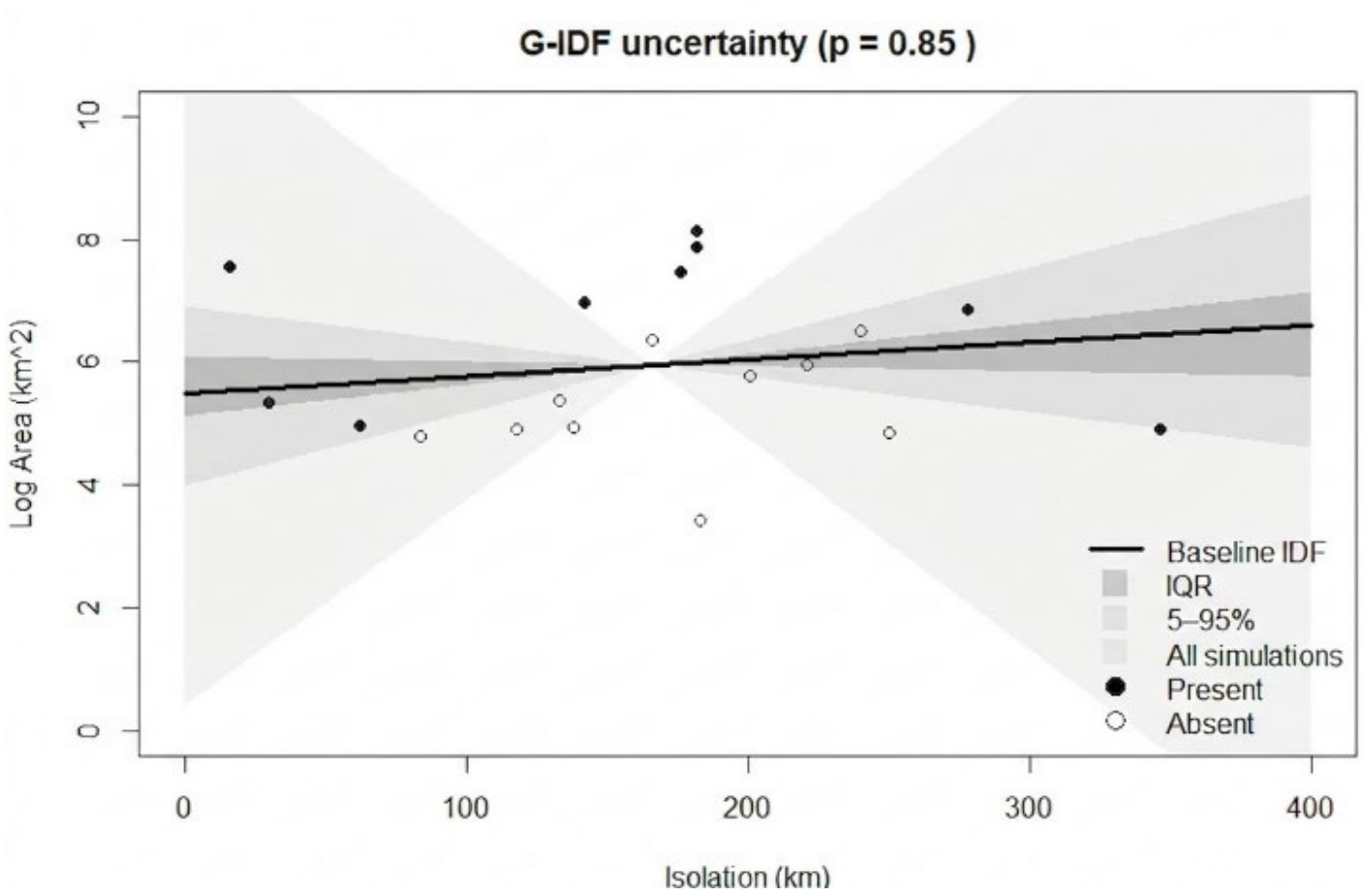
G-IDF uncertainty (p = 0.85)
Log Area (km^2)
Isolation (km)
Baseline IDF
IQR
5–95%
All simulations
Present
Absent

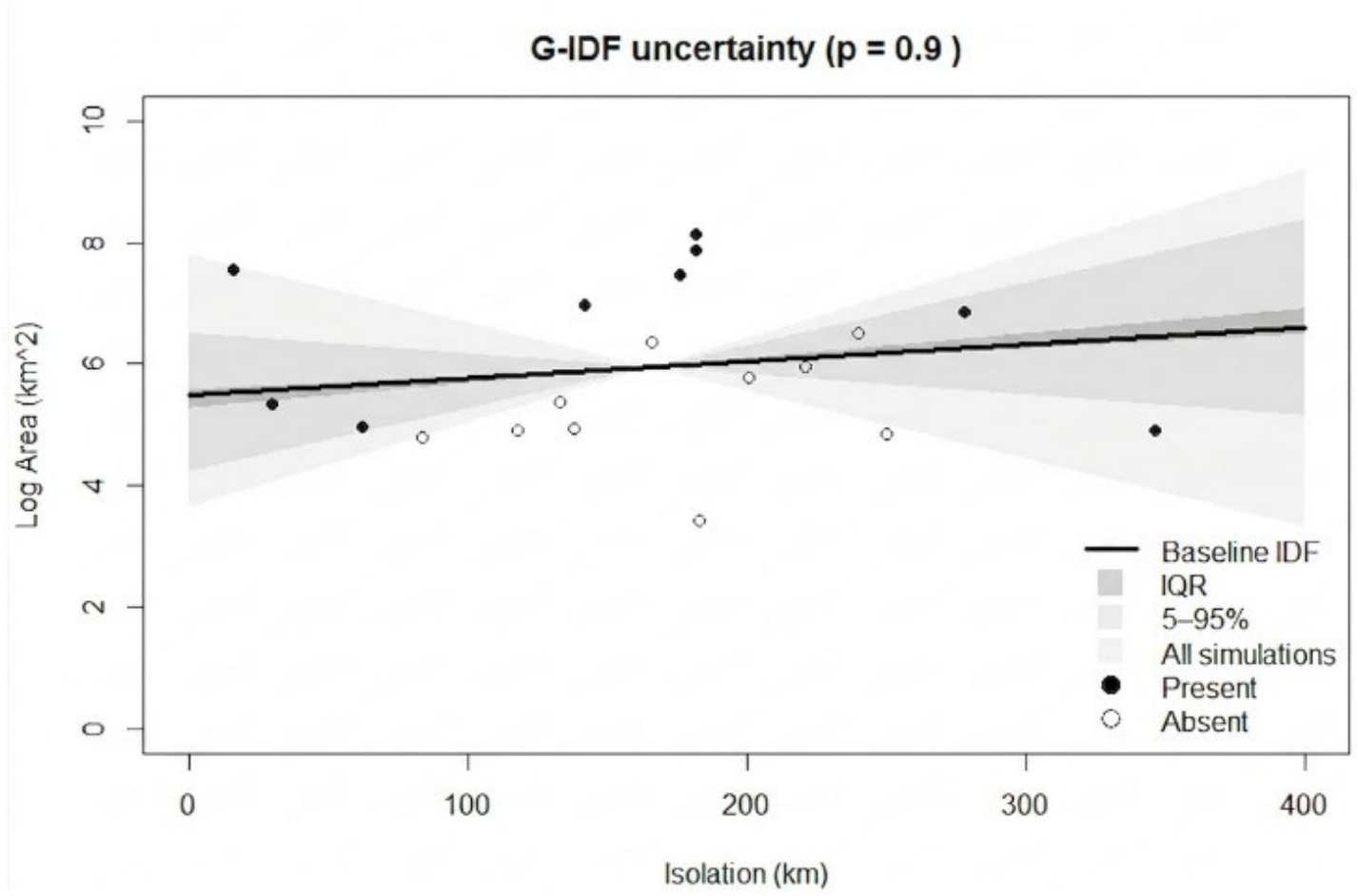
G-IDF uncertainty (p = 0.9)
Log Area (km^2)
Isolation (km)
Baseline IDF
IQR
5–95%
All simulations
Present
Absent

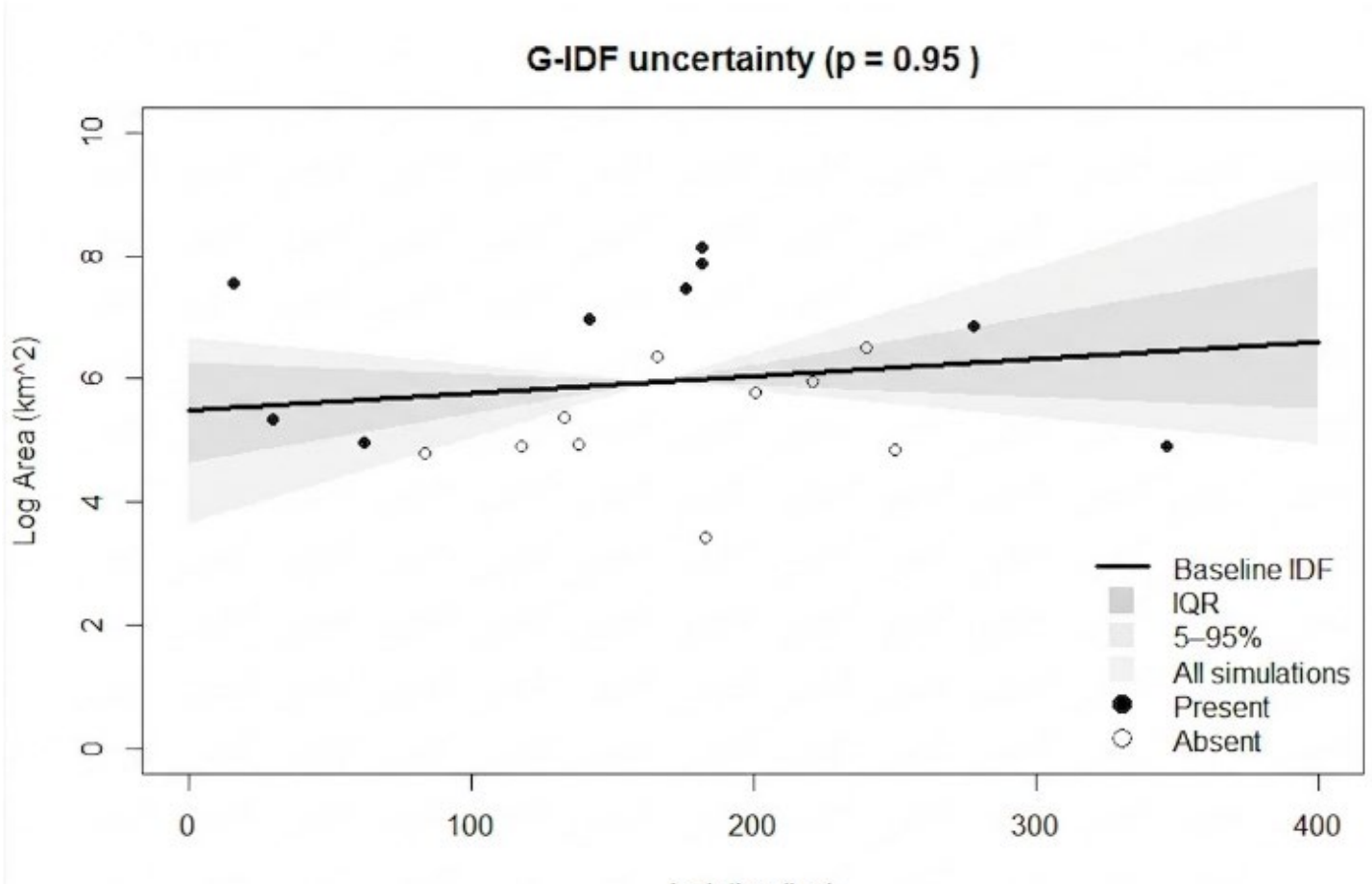
G-IDF uncertainty (p = 0.95)
Log Area (km^2)
Isolation (km)
Baseline IDF
IQR
5–95%
All simulations
Present
Absent

Figure S6

Distribution of E-IDF slope values from 200 simulations per level of detection probability.

Vertical dashed lines indicate the baseline slope (no detection error).

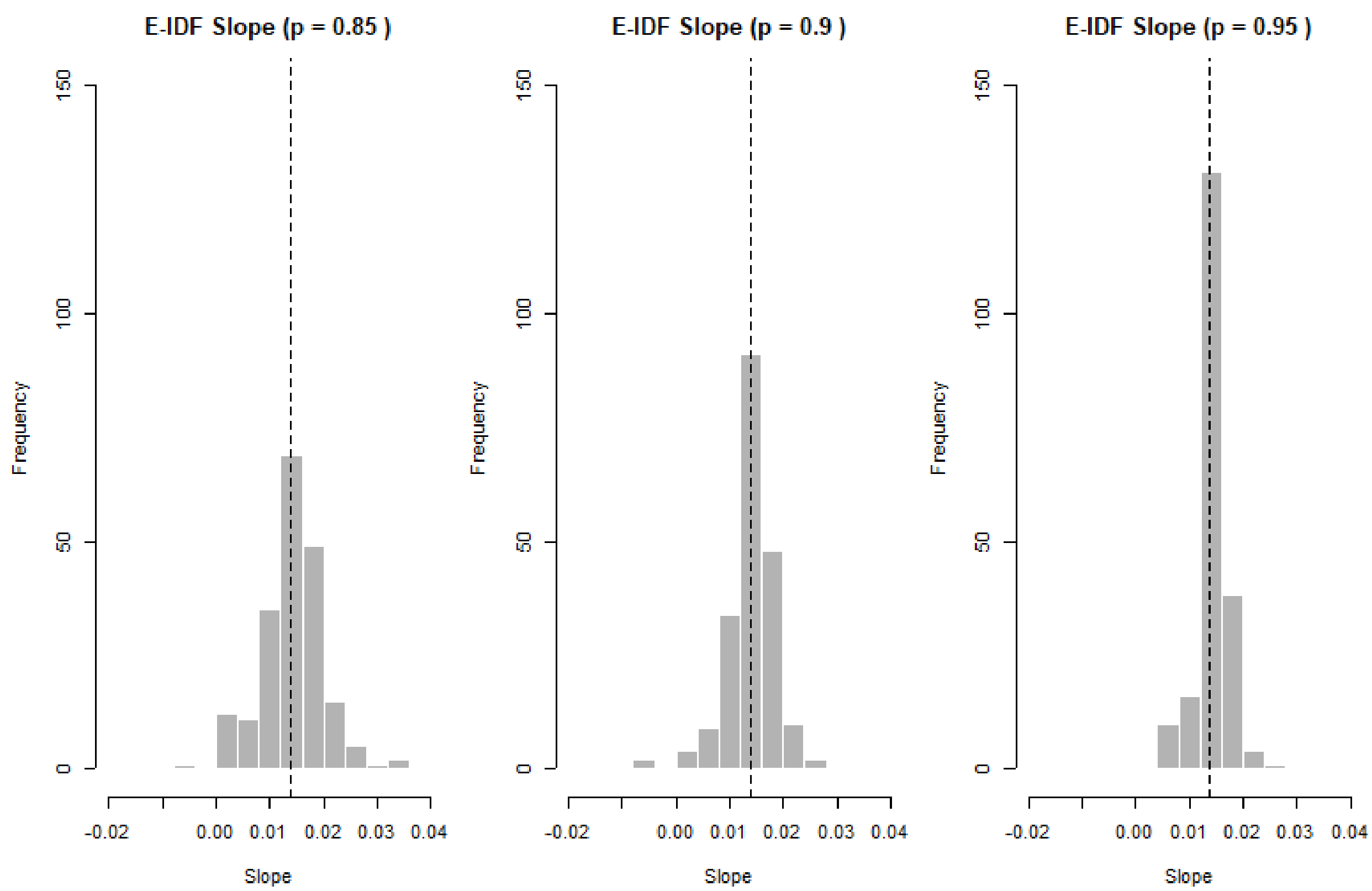

Figure S7

Distribution of G-IDF slope values from 200 simulations per level of detection probability.

Vertical dashed lines indicate the baseline slope (no detection error).

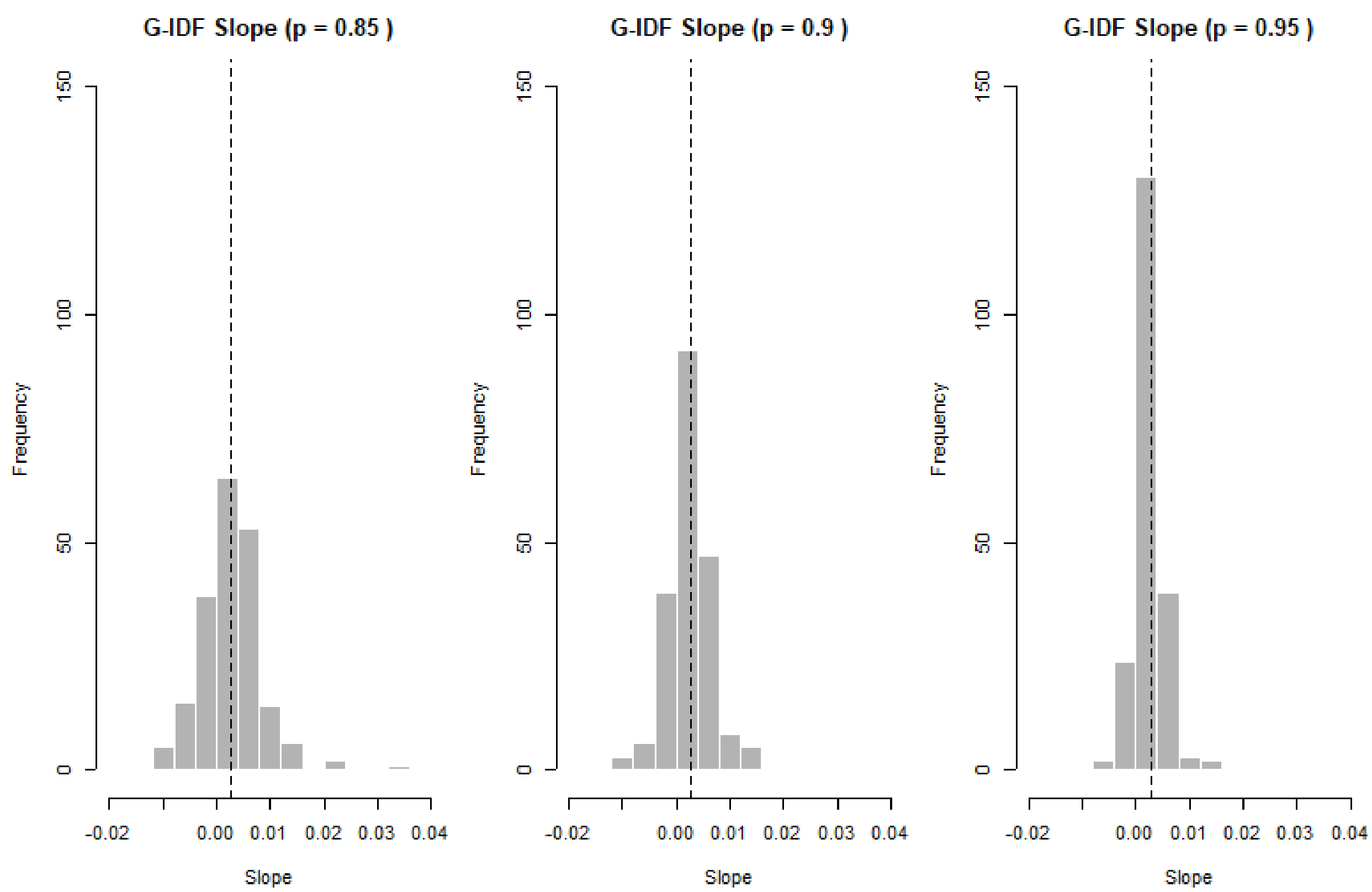

Figure S8

Distribution of E-IDF intercept values from 200 simulations per level of detection probability.

Vertical dashed lines indicate the baseline intercept (no detection error).

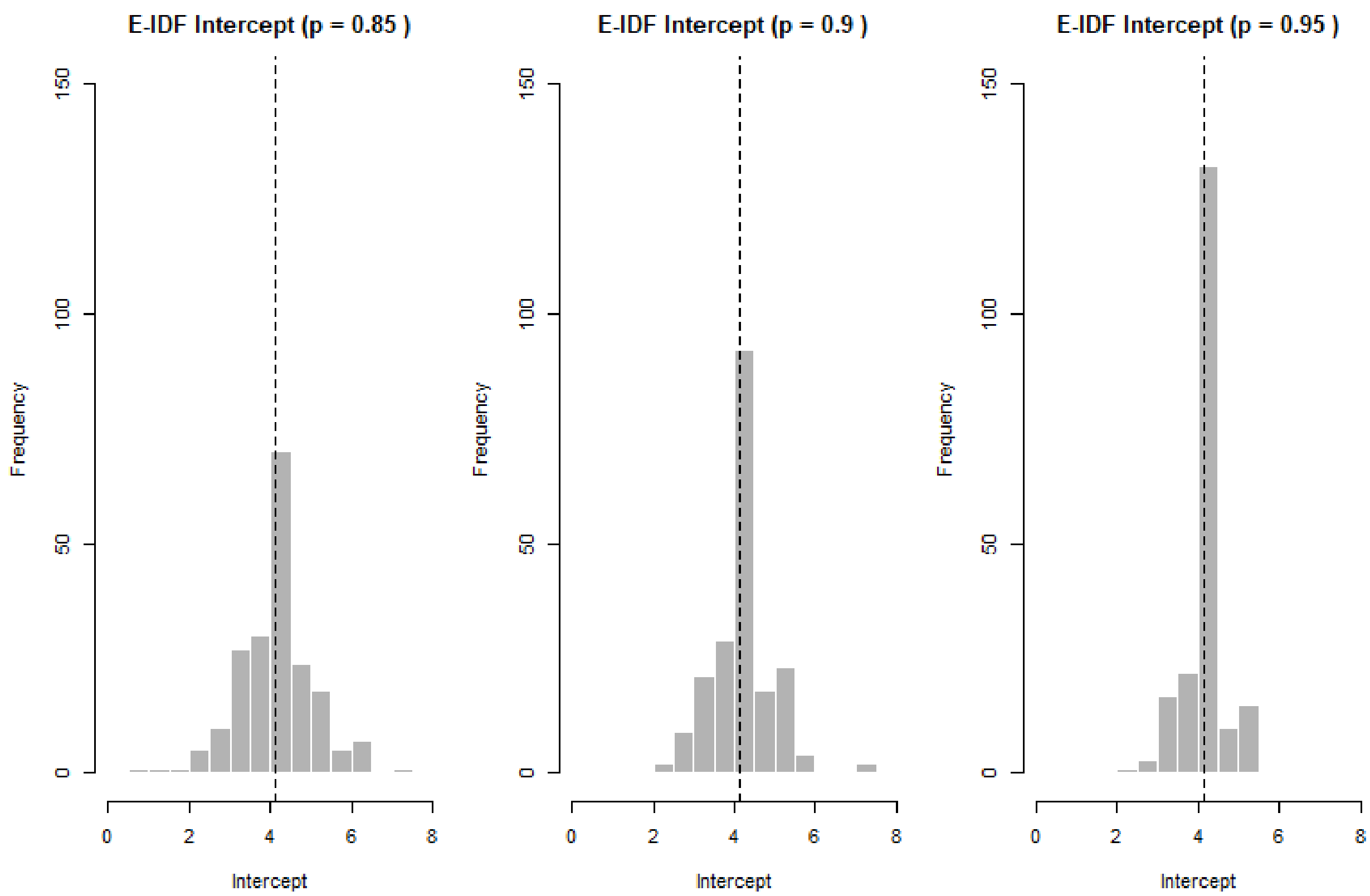

Figure S9

Distribution of G-IDF intercept values from 200 simulations per level of detection probability.

Vertical dashed lines indicate the baseline intercept (no detection error).

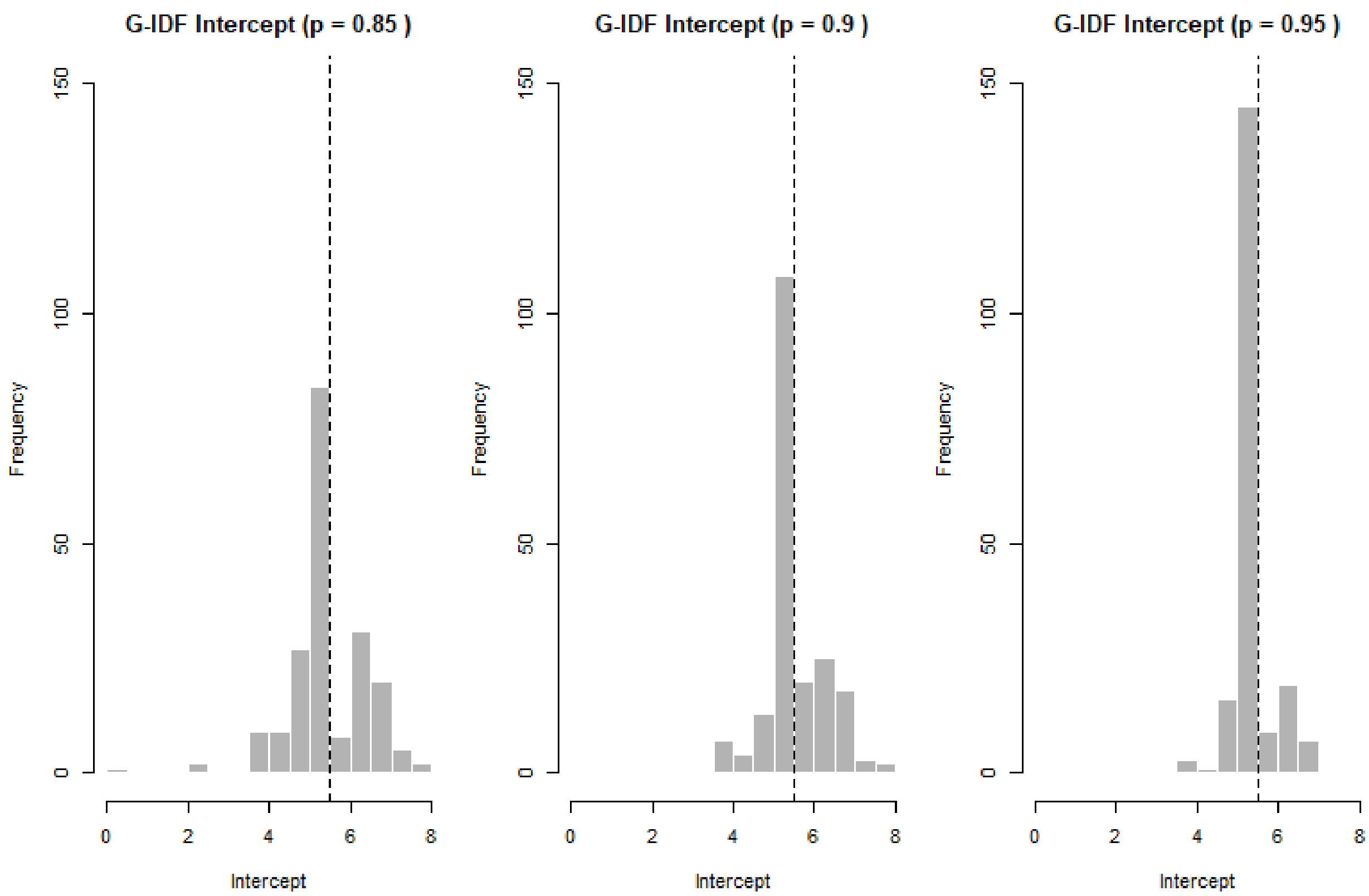

Figure S10

Paired plotting of slopes for G-IDF vs. E-IDF under each level of detection probability using the same degraded data for each replicate. Each point represents one simulation replicate. The dashed line indicates 1:1 equivalence. Points above the line indicate simulations where the E-IDF produced a higher slope than the G-IDF. Note the greater variation at lower levels of detection probability (with higher false-absence errors).

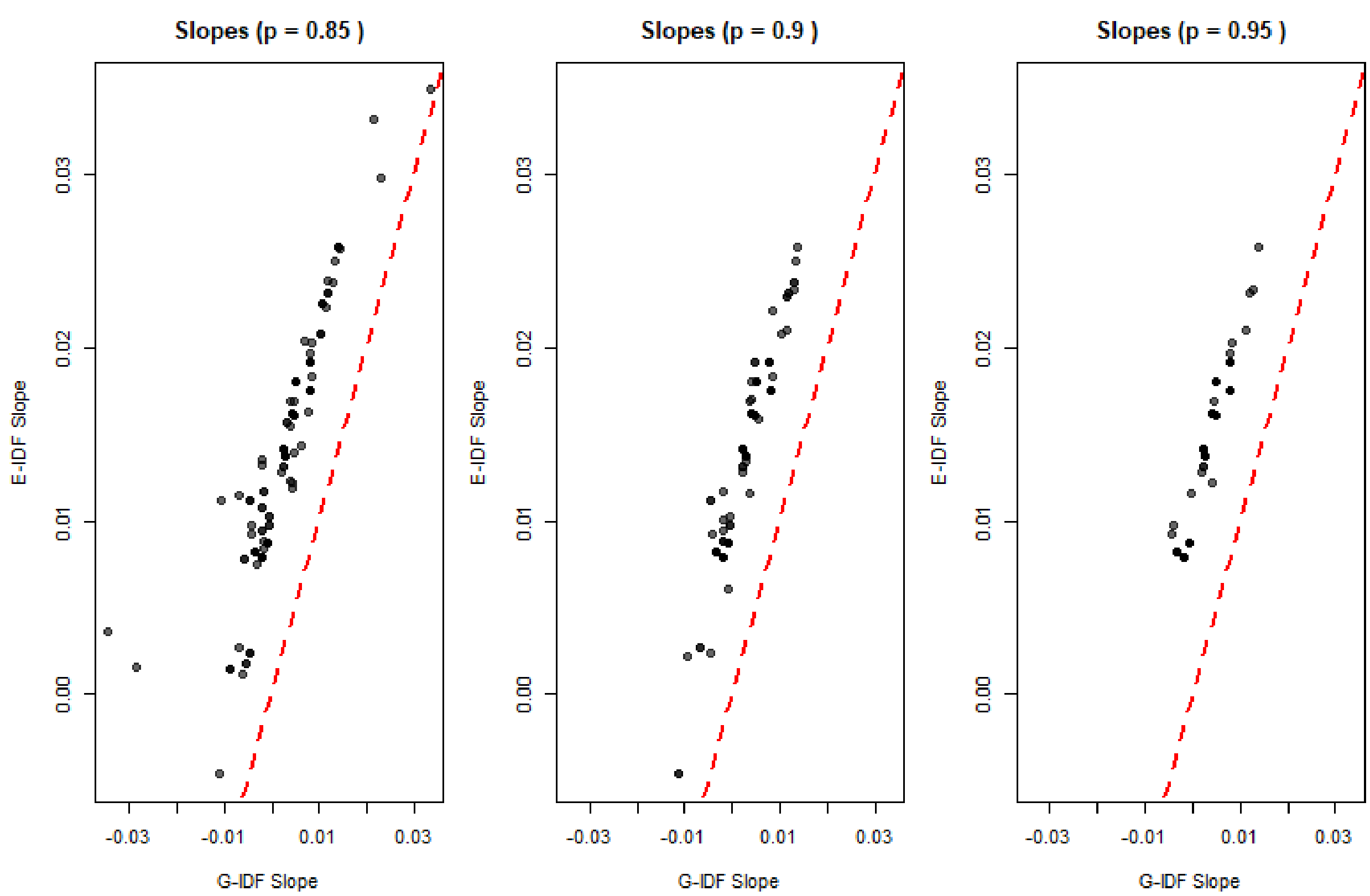

Figure S11

Paired comparisons of intercepts for G-IDF vs. E-IDF under each level of detection probability using the same degraded data for each replicate. Each point represents one simulation replicate. The dashed line indicates 1:1 equivalence. Points below the line indicate simulations where the G-IDF produced a higher intercept than the E-IDF. Note the greater variation at lower levels of detection probability (with higher false-absence errors).

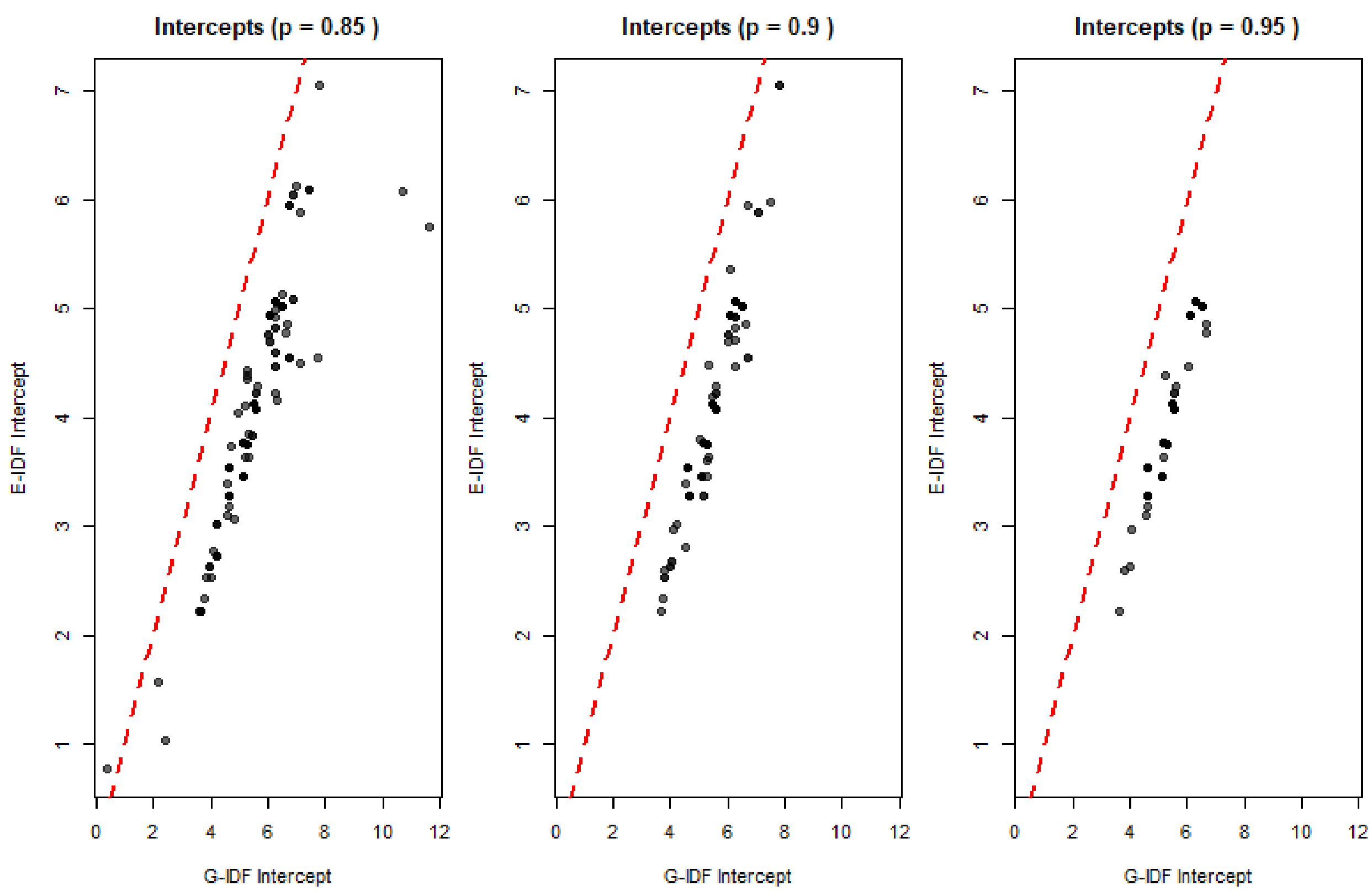

Supplement: Supplementary file 1 [file ECY-107-e70541-s001.pdf]
